# Prospective Molecular Profiling of Canine Cancers Provides a Clinically Relevant Comparative Model for Evaluating Personalized Medicine (PMed) Trials

**DOI:** 10.1371/journal.pone.0090028

**Published:** 2014-03-17

**Authors:** Melissa Paoloni, Craig Webb, Christina Mazcko, David Cherba, William Hendricks, Susan Lana, E. J. Ehrhart, Brad Charles, Heather Fehling, Leena Kumar, David Vail, Michael Henson, Michael Childress, Barbara Kitchell, Christopher Kingsley, Seungchan Kim, Mark Neff, Barbara Davis, Chand Khanna, Jeffrey Trent

**Affiliations:** 1 Comparative Oncology Program, Center for Cancer Research, National Cancer Institute, Bethesda, Maryland, United States of America; 2 Van Andel Research Institute, Grand Rapids, Michigan, United States of America; 3 Translational Genomics Research Institute (TGen), Phoenix, Arizona, United States of America; 4 Colorado State University, College of Veterinary Medicine, Fort Collins, Colorado, United States of America; 5 Clinical Reference Laboratory, Lenexa, Kansas, United States of America; 6 University of Wisconsin-Madison, School of Veterinary Medicine, Madison, Wisconsin, United States of America; 7 University of Minnesota, College of Veterinary Medicine, St. Paul, Minnesota, United States of America; 8 Purdue University, School of Veterinary Medicine, West Lafayette, Indiana, United States of America; 9 Michigan State University, College of Veterinary Medicine, East Lansing, Michigan, United States of America; Duke-NUS, Singapore

## Abstract

**Background:**

Molecularly-guided trials (i.e. PMed) now seek to aid clinical decision-making by matching cancer targets with therapeutic options. Progress has been hampered by the lack of cancer models that account for individual-to-individual heterogeneity within and across cancer types. Naturally occurring cancers in pet animals are heterogeneous and thus provide an opportunity to answer questions about these PMed strategies and optimize translation to human patients. In order to realize this opportunity, it is now necessary to demonstrate the feasibility of conducting molecularly-guided analysis of tumors from dogs with naturally occurring cancer in a clinically relevant setting.

**Methodology:**

A proof-of-concept study was conducted by the Comparative Oncology Trials Consortium (COTC) to determine if tumor collection, prospective molecular profiling, and PMed report generation within 1 week was feasible in dogs. Thirty-one dogs with cancers of varying histologies were enrolled. Twenty-four of 31 samples (77%) successfully met all predefined QA/QC criteria and were analyzed via Affymetrix gene expression profiling. A subsequent bioinformatics workflow transformed genomic data into a personalized drug report. Average turnaround from biopsy to report generation was 116 hours (4.8 days). Unsupervised clustering of canine tumor expression data clustered by cancer type, but supervised clustering of tumors based on the personalized drug report clustered by drug class rather than cancer type.

**Conclusions:**

Collection and turnaround of high quality canine tumor samples, centralized pathology, analyte generation, array hybridization, and bioinformatic analyses matching gene expression to therapeutic options is achievable in a practical clinical window (<1 week). Clustering data show robust signatures by cancer type but also showed patient-to-patient heterogeneity in drug predictions. This lends further support to the inclusion of a heterogeneous population of dogs with cancer into the preclinical modeling of personalized medicine. Future comparative oncology studies optimizing the delivery of PMed strategies may aid cancer drug development.

## Introduction

Novel approaches are needed to improve outcomes for cancer patients. In the last decade, advances in biological platforms and investigative tools have permitted the molecular characterization of cancer in a clinically relevant setting. Indeed, the field of personalized medicine (PMed) represents the integration of genomic, proteomic and epigenetic data in the characterization of a patient's cancer [1], [2], [3], [4]. Goals of personalized medicine are to reveal unique disease drivers or susceptibilities, potential toxicities, and resistance profiles and develop patient-specific therapeutic interventions. Despite the promise in this approach, many gaps remain in the determination of best practices, the feasibility of real-time molecular profiling of patient samples in support of therapeutic decision-making, and the actual clinical benefit of these time-consuming and costly techniques.

Molecular features of cancers have been the basis for selecting specific treatments of patients for over a decade. Initial approaches were candidate-based, such as the use of imatinib (Gleevec) for acute myeloid leukemias harboring *BCR-ABL* gene translocations, HER2Neu positive breast cancer treatment with trastuzumab (Herceptin), and, prior to this, tamoxifen in ER/PR positive breast cancer patients [5], [6]. Such approaches represent some of the earliest forms of molecularly guided therapy. In its current form, PMed has evolved to represent a large-scale non-candidate based assessment of a given cancer across the whole genome with greater pharmacopeia coverage, rather than queries of specific candidate analytes for a single disease-drug context [7], [8]. It encompasses a series of high throughput analyses such as gene expression, whole-genome sequencing, whole exome-sequencing and epigenetic assessments aimed to detail somatic and inherited mutations in individual patients and their tumors. However, genome-wide surveillance is complex and does not necessarily lead to a single or defined intervention. Sophisticated mathematical algorithms are needed to integrate these large pools of molecular data and then match or identify appropriate or reasonable therapeutic approaches. Examples of non-candidate PMed clinical studies have been reported. The Bisgrove trial, conducted by Von Hoff et al., treated 66 patients with refractory and metastatic cancers with regimens chosen through immunohistochemical and gene expression profiling of each patient's tumor in conjunction with heuristic biomarker rules based upon literature evidence [9]. Progression free survival (PFS) improved compared to the immediate previous regimen in 27% of patients [9]. Tsimberidou et al. described the benefits of molecularly tailored therapy over non-molecularly matched therapy with higher overall response rates (27% v. 5%), longer time to treatment failure (median 5.2 v. 2.2 months), and improved overall survival (median 13.4 v. 9 months) in Phase I studies [10]. Other studies have similarly demonstrated the feasibility and potential utility of PMed approaches in a variety of clinical settings[11] [12]. The early successes in proof-of-concept trials with human patients emphasize the need to optimize various aspects of PMed for broader clinical application. Examples of areas in need of optimization include the improvement of sample collection and processing techniques, the definition of molecular features of patient samples, and the application of mathematical algorithms to integrate these large pools of molecular data to model relevant therapeutic approaches. The quantity and diversity of available data coupled with differences in processing algorithms can make it difficult to determine how to prioritize the links between molecular targets and therapeutic agents [13]. Indeed, comparisons between algorithms that seek to match targets with therapeutics are needed. Conventional preclinical models of cancer are not characterized by the individual-to-individual heterogeneity seen in human cancers. As such there is limited opportunity to use these preclinical models to effectively optimize and translate components of PMed. Furthermore, it is unlikely that such optimization of PMed can be accomplished in human trials alone.

Comparative oncology is most often used to describe the study of cancer biology and therapy in pet animals that naturally develop cancer [14], [15], [16]. The heterogeneity and complexity of cancer in the pet dog population and within cohorts of dogs with the same histological diagnoses is well suited for modeling PMed. The public availability of a progressively annotated canine genome and the advent of high throughput genomic techniques for the dog has enabled comparative oncology to describe canine cancer biology and define potential therapeutic targets in many of the same ways as human cancers [17]. In addition, strong cancer breed predilections support ‘breed-based’ germ-line discoveries that may streamline the definition of specific cancer targets as “drivers” of a cancer event. Since comparative oncology modeling does not require up-front treatment with specific cancer treatment regimens, novel therapeutic agents can be offered through clinical trials at any stage in cancer presentation. Compressed disease progression times in pet dogs with cancer allow for the evaluation of a variety of PMed interventions against longitudinal endpoints of cancer progression in ways not possible in the human clinic. Finally comparative oncology randomized control trials can be conducted in the newly-diagnosed, adjuvant (i.e. minimal residual disease) and metastatic settings, evaluating the utility of PMed drug selection and algorithm prediction across a range of clinical scenarios.

To begin to realize these opportunities to model PMed strategies, a proof of concept study was conducted through the Comparative Oncology Trials Consortium (COTC) to determine if the collection and analysis of tumor samples from dogs with cancer, within a PMed framework, could be completed in a time period (<1 week) considered feasible for implementation in a future therapeutic trial. Tumor biopsies across multiple histologies and in cohorts of canine bladder transitional cell carcinoma (TCC), lymphoma, and melanoma were collected and quality assurance/control measures applied to each step in the process of generating molecular data to support a PMed derived therapeutic report. The results revealed that high-quality, prospective tumor collections, and large-scale target/drug identification studies in canine cancers are feasible. As observed in human PMed trials, tumor gene expression signatures in dogs cluster by cancer type, whereas the personalized drug reports were uniquely patient defined. Data from this study serves as rationale to now include dogs with spontaneous cancers in the advancement and optimization of PMed for human patients.

## Results

### Study Enrollment

The study design (**Table 1:** **Study Schedule**) provided for prospective tumor collection and real-time molecular profiling in dogs with cancer. A total of 31 dogs were enrolled and assigned to one of four cohorts. The first cohort was open to all cancer types (n = 15 enrolled, 10 samples passed QA/QC), while the remaining three cohorts were breed and/or cancer type specific. The cancer type specific cohorts included Scottish terriers with bladder transitional cell carcinoma (n = 5 enrolled, 4 passed QA/QC), golden retrievers with lymphoma (n = 5 enrolled, 5 passed QA/QC), and American cocker spaniels with melanoma. The melanoma cohort was opened to all breeds after three months to enhance accrual (n = 6 enrolled, 5 passed QA/QC). Age (range: 5.1–13.4 years, median 9.7 years), sex (18 spayed females, 1 intact female, 9 castrated males, 3 intact males) and breed (5 mixed-breed and 26 purebred) were recorded variables for all dogs enrolled (**Table 2:** **Study Cohorts**). The trial opened on May 11, 2011 and closed on October 19, 2011 upon achieving its accrual goals. There were no significant adverse events reported (according to VCOG-CTCAE convention) [18].

**Table 1:** Study Schedule.

ACTION	ELIGIBILITY	DAY 1
Tumor measurements (caliper or US measurement (cm))	X	
Physical Exam	X	X
Digital photo of tumor		X
Serum, plasma collection		X
Tumor Biopsy (frozen and formalin)		X
Buccal/saliva sample collection		X

**Table 2:** Study Cohorts.

COHORT	CASES ENROLLED	CASES PASSING QA/QC
Scottish Terriers with Transitional Cell Carcinoma	5	4
Golden Retrievers with Lymphoma	5	5
Various breeds with Melanoma	6	5
Open histology (any breed/histology)	15	10

### Quality Assessment/Quality Control measures were successful in defining high quality tumor samples for expression analysis

Histopathology quality assurance and control (QA/QC) assessment of all biopsies were performed by one pathologist (EJE). Twenty-four of 31 cases enrolled (77%) passed QA/QC with an average tumor surface area of 75–100%, tumor nuclei of 75–100%, and necrosis < or equal to 10%. (**Table 2:** **Study Cohorts**) Reasons for histopathology QA/QC failures included samples with too little viable tumor, high degree of necrosis, small sample size, or non-cancer diagnosis (**Table 3:** **Reasons samples failed QA/QC**).

**Table 3:** Reasons samples failed QA/QC.

DISEASE TERM	% TUMOR NUCLEI (>75% PASS)	% TUMOR NECROSIS (<20% PASS)	TOTAL YIELD ≥20 NG (IN 14 UL) (PASS)	NANODROP 260/280 ≥1.8 (PASS)	BIOANALYZER RIN ≥8.0 (PASS)	REASON FOR SAMPLE FAILURE
Bladder transitional cell carcinoma-trigonal (0204)	25–50% Fail	<20%	3.91 ng/ul	3.76	N/A	Sample too small for analysis
Lymphoma (0503)	75–100%	>20% Fail	3092.04 ng/ul	2.03	9.40	High degree of sample necrosis
Osteosarcoma (appendicular) (0506)	0–24% Fail	<10%	69.99 ng/ul	2.08	9.60	Too little viable tumor to analyze
Mast cell tumor (0507)	75–100%	<10%	614.62 ng/ul	2.10	2.60 Fail	Poor quality RNA due to abundant connective tissue
Melanoma-mucosa/mandible (0502)	0–24% Fail	<10%	536.99 ng/ul	2.08	8.2	Sample too small for analysis
Lymphoma (1301)	0–24% Fail	N/A	16.67 ng/ul	1.93	9.60	Sample too small for analysis
Histiocytic Sarcoma (1302)	0–24%	N/A	657.76 ng/ul	2.12	9.80	Non-cancer diagnosis. Histopathology was panniculitis

**Figure 1:**
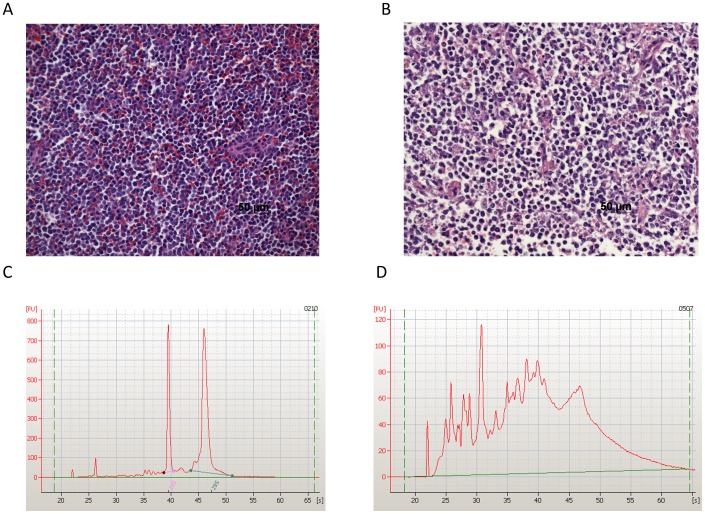
Histopathology and RNA quality assurance and control measures were successful in procuring high quality canine tumor samples. Formalin-fixed, paraffin-embedded tumor biopsy samples were sectioned, paraffin embedded, and H&E stained for light microscopic evaluation. A single board-certified veterinary pathologist (EJE) assessed % tumor surface area, % tumor nuclei and % tumor necrosis to determine their quality prior to molecular profiling. Images of representative H&E images are shown: **A.** Sample 0209, a golden retriever with lymphoma, passed QA/QC. (Tumor 75–100%, necrosis <10%), while **B.** sample 0503, a beagle with lymphoma, failed QA/QC (Tumor 75–100%, necrosis >20%). Biopsies that failed to pass QA/QC in any category were excluded from subsequent analysis. Additionally RNA isolation was performed for all enrolled cases (n = 31) at a CLIA certified laboratory. RNA was extracted from Tumor A biopsy samples. Quality measures included quantity (total yield >20 ng) and integrity (A_260_/A_280_>1.8, RIN>8.0) measured by Nanodrop and Agilent Bioanalyzer. Electropherograms from cases **C.** 0210 and **D.** 0507 are depicted. Sample 0210, an oral melanoma, passed RNA QA/QC while sample 0507, a mast cell tumor, failed QA/QC (poor quality RNA due to a large connective tissue component).

RNA isolation and QA/QC assessment was performed for all enrolled cases (n = 31) at a Clinical Laboratory Improvement Amendments (CLIA) accredited facility (Clinical Reference Laboratory, Lenexa, KS) to ensure quality laboratory testing. QA/QC standards defined here have been previously used for the conduct of human tissue processing and clinical trials (http://wwwn.cdc.gov/clia) [19]. Quality measures for RNA isolation were quantity (total yield >20 ng) and integrity (A_260_/A_280_≥1.8, RIN≥8.0) measured by Nanodrop and Agilent Bioanalyzer respectively. Thirty of the 31 cases (96.78%) passed RNA QA/QC. One sample (0507) failed QA/QC due to poor RNA quality (low RIN score = 2.60), likely due to its abundant connective tissue component (**Figure 1** **and** **Table 3:** **Reasons samples failed QA/QC**). Finally, cDNA was then amplified for all remaining samples. Quality control for amplified cDNA included isolation quantity (total yield ≥5 ug) and integrity (260/280≥1.8); all 30 samples passed cDNA assessment.

Each case underwent the above described histopathologic and RNA/cDNA evaluations. Samples (n = 24/31) that passed all stages of QA/QC were analyzed for gene expression on an Affymetrix platform (Canine Genome v 2.0). Common reasons for QA/QC failures were small specimens or specimens with an inadequate amount of viable tumor present (**Table 3:** **Reasons samples failed QA/QC**). These results are consistent with those of tissues collected for human PMed trials.

### Bioinformatics analyses utilized genomics data to generate individual patient personalized medicine reports within a clinically relevant time frame

**Figure 2:**
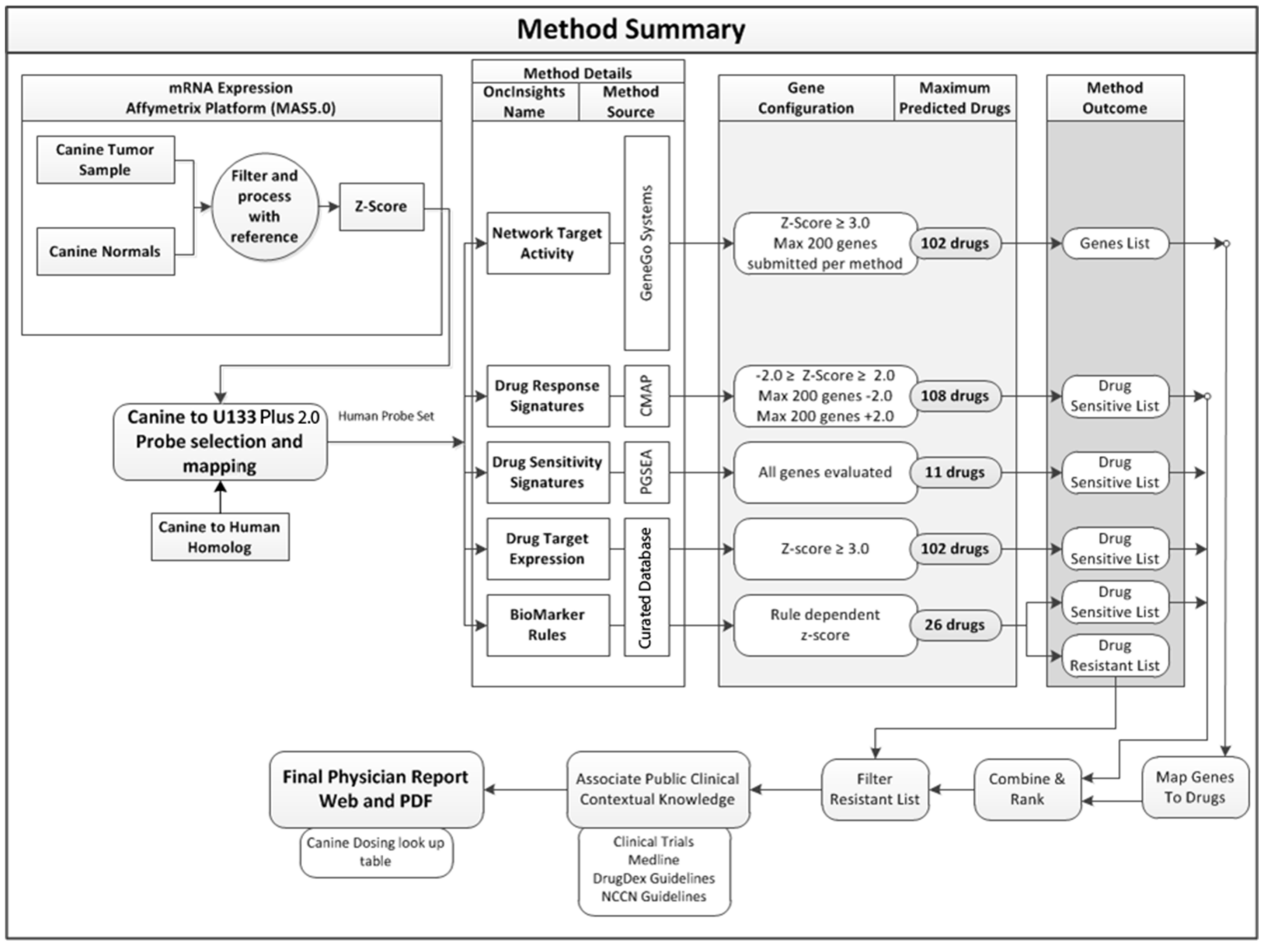
Bioinformatic analysis defines the platform for PMed report generation. Gene expression data from each tumor was compared to a reference sample set (canine normal tissue compendium, GSE20113 from Gene Expression Omnibus) to obtain a relative gene expression profile. Each gene probeset was represented by a z-score depicting its expression in the tumor in terms of the number of standard-deviations from the mean expression in the reference set. In the iteration of the PMed tools used in this study, data were analyzed by six distinct predictive methodologies (Drug Target Expression, Drug Response Signatures, Drug Sensitivity Signatures, Network Target Activity, Biomarker-Based-Rules-Sensitive, Biomarker-Based-Rules-Insensitive) to identify (or exclude in the case of biomarker resistant rules) potential agents for consideration. All predictions were based on the conversion of canine genomic data into human homologs (for both patient tumor samples and the reference set of normal tissues) prior to the application of the specific algorithms that rely exclusively on human knowledge and/or empirical drug screens using human cell lines (see Methods). While individual patient tumor PMed report generation and distribution was the final step in this process, this specific study did not have therapeutic intent and drug prescription was not performed.

Gene expression data from each tumor was compared to that of a reference gene set to define a relative gene expression profile. The reference set consisting of forty normal canine tissues was used to estimate variance in gene expression across normal physiology [20]. Each gene probeset was represented by a z-score depicting its tumoral expression in terms of the number of standard-deviations from the mean expression of that probe set in the reference data. Genes with a positive z-score in the tumor were thereby over-expressed whereas those with a negative z-score were under-expressed. Expression data was then analyzed by six predictive methodologies (Drug Target Expression, Drug Response Signatures, Drug Sensitivity Signatures, Network Target Activity, Biomarker-Based-Rules-Sensitive, Biomarker-Based-Rules-Insensitive) to identify potential therapeutic agents for consideration (**Figure 2**) according to a previously-elucidated workflow [19]. Drug sensitivity was ranked by z-score and p values were transformed (−log(*p*)) and reported individually for each specific algorithm then summated (sum of (−log(*p*) across algorithms) to provide an overall prediction of drug selection. A summary table and drug method comparison defined the top selected agents (**Table S1**). The summary gives more weight to drugs suggested by more than one algorithm. PMed reports were not intended to be used therapeutically in this pilot study, although their timely generation demonstrated the bioinformatics feasibility to use the dog as a model for future PMed clinical trials.

**Figure 3:**
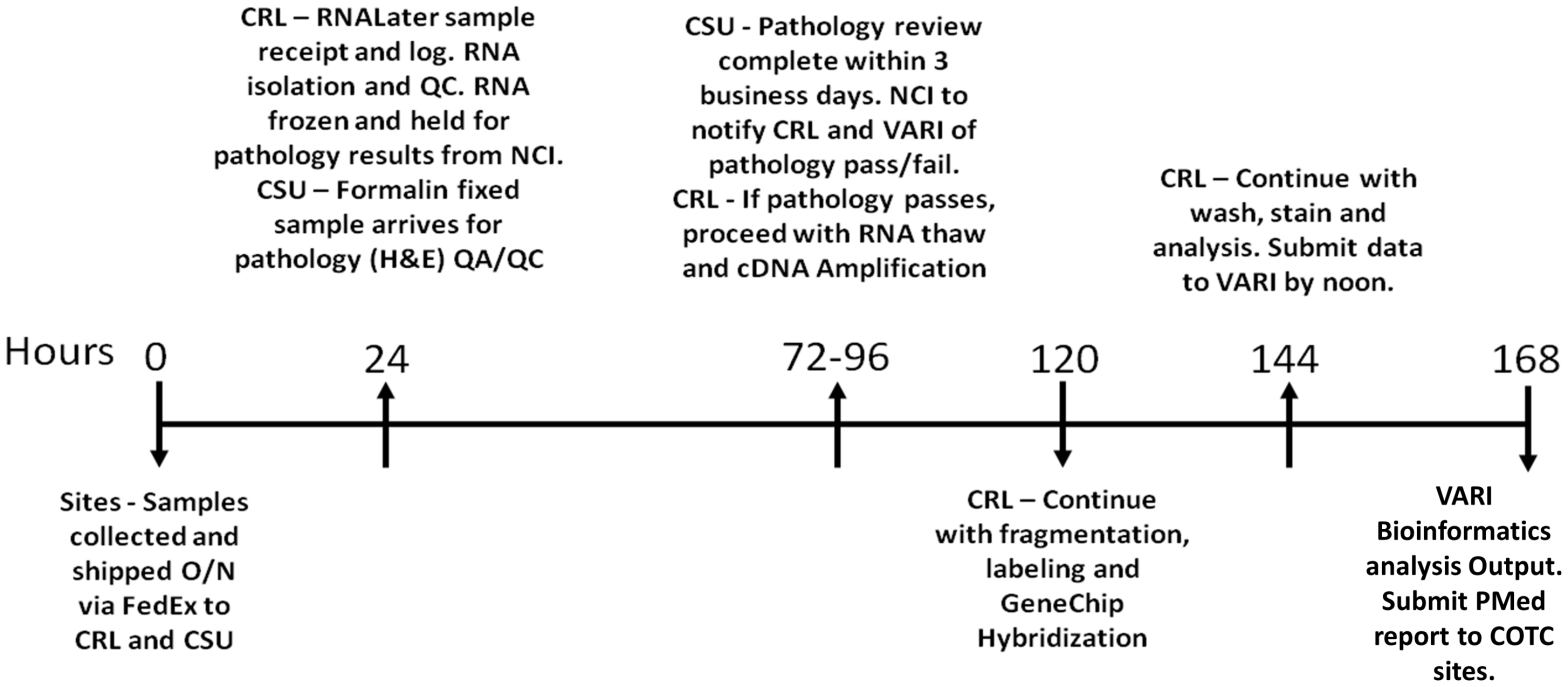
Expected clinical turnaround time for canine tumor sample collection, processing, expression, bioinformatic analysis and PMed report delivery. The graphic defines the prospective timeline of key steps in the process of sample collection, shipment, histopathology and RNA quality assurance and control assessments, expression profiling and PMed report generation. Samples were biopsied at their clinical COTC site, sent to histopathology and CLIA labs for parallel sample and RNA QA/QC, Affymetrix gene expression analysis performed, and the derived genomic data sent to the Van Andel Research Institute for bioinformatics evaluation and PMed report generation. Minimum feasible turnaround time for sample analysis was described prospectively as 7 business days (168 hours), however all cases were completed in 4.85 days (116.46 hours). The process was successful in defining high quality tissues for molecular analysis and will be used in future canine PMed comparative studies.

The minimum feasible clinical time line (time from sample ascertainment and shipment from COTC site to completed PMed report returned to attending clinician) was defined prior to study initiation as 168 business hours (< or equal to 7 days) (**Figure 3**). Turnaround time for all cases was faster than projected. It was less than 5 business days (n = 24, 116.5 business hours (4.85 days) and 168.46 total total hours (7.01 days)). (**Table 4**: **Clinical Turn Around Time**). Clinical turnaround time for case 0508 (TCC) was an outlier (completed in 212 business hours). The expression data was generated in 91 business hours (3.79 days), but there was a delay in sending the PMed report to investigators. Its inclusion in the analysis did not impact the study conclusions. Overall the turnaround for sample analyses fit a relevant clinical window for future comparative oncology trials to model human PMed advancements.

**Table 4:** Clinical turn around time.

COHORT	PATIENT NUMBER	TOTAL TIME IN BUSINESS HOURS (DAYS)	TOTAL TIME IN HOURS
Open histology	10	114.50 (4.77)	174.50
Lymphoma	5	117.00 (4.88)	165.00
TCC	4	116.75 (4.86)	158.75
Melanoma	5	119.60 (4.98)	167.60
All Cases*	24	116.46 (4.85)	168.46

*Clinical turnaround time for case 0508 (TCC) was an outlier (completed in 212 business hours).

The expression data was generated in 91 business hours (3.79 days) but there was a delay in the PMed report being sent to investigators. Overall the turn around for sample analysis fits a clinical window and its inclusion in the analysis did not impact the study conclusions.

### Canine tumor samples clustered by cancer type but drug reports were patient specific

**Figure 4:**
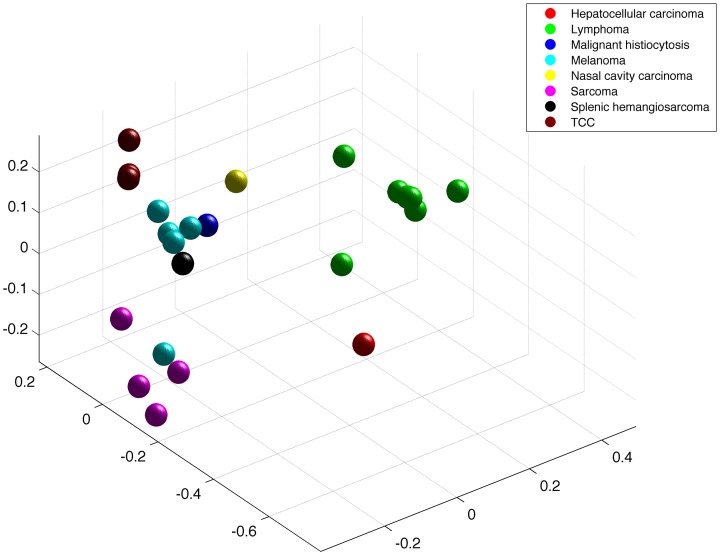
Cancer type defines canine tumor gene expression signatures. Multidimensional scaling (MDS) coordinates were generated using individual tumor gene (mRNA) expression z-scores to define relationships within the dataset. Tumor gene expression clustered by tumor type. Additionally, histologic categories share genomic signatures, with carcinomas (bladder TCC, nasal carcinoma, hepatocellular carcinoma (HCC)), mesenchymal (soft tissue sarcomas, hemangiosarcoma, histiocytic sarcoma, melanoma), and round cell (lymphoma) tumors clustering together in subgroups.

To characterize the utility of the resultant canine tumor expression data for future therapeutic consideration, clustering analysis was performed. Multidimensional scaling (MDS) coordinates were generated using individual tumor gene (mRNA) expression and drug prediction scores. Consistent with others efforts using MDS and principal component analysis (PCA) of human tumors, gene expression in the dog tumors clustered by cancer type (**Figure 4**). As expected, broad histologic categories shared genomic signatures, with carcinomas (bladder TCC, nasal carcinoma, hepatocellular carcinoma (HCC)), mesenchymal (soft tissue sarcomas, hemangiosarcoma, histiocytic sarcoma, melanoma), and round cell (lymphoma) tumor samples clustering in subgroups. The single HCC sample was an outlier due to liver specific genes being highly expressed with high variance relative to other samples. Breed was analyzed as an independent variable in tumor gene expression but did not influence clustering (**Figure S1**). Both pure bred and mixed breed dog samples were grouped by histologic description.

**Figure 5:**
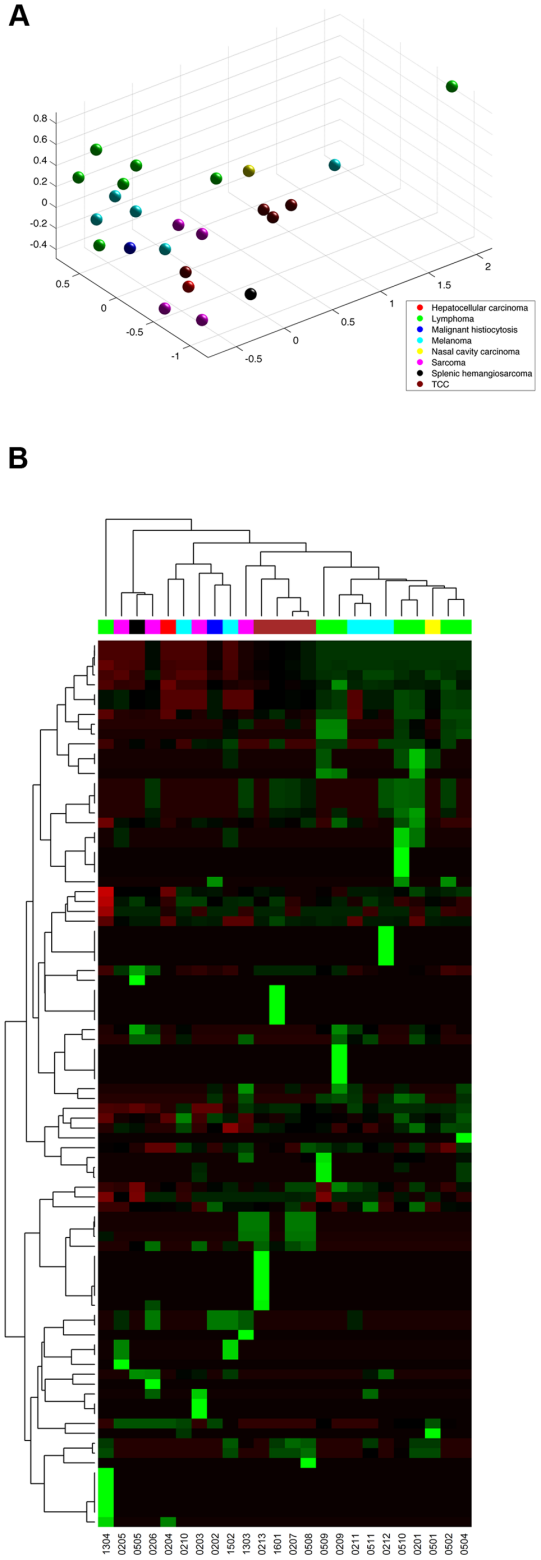
Drug prediction scores define individual tumor predicted drug susceptibilities. **A.** MDS analysis shows nested PMed summary drug scores cluster individual samples by drug susceptibility. There was a weak association of drug calls with cancer type, but clear heterogeneity in drug prediction even within a single cancer type (histology). **B.** A heat map of targeted and conventional agent sensitivity across each patient sample. Individualized drug predictions based on tumor characteristics support the use of PMed drug prescription in future comparative oncology studies.

The second phase of MDS analysis used total nested PMed drug score, a summation of individual method scores, to cluster individual samples by drug susceptibility (**Figure 5**). The drug pool available for this analysis included 184 FDA approved agents. There was a weak association of drug calls with tumor type, but also clear heterogeneity in drug prediction even within a defined cancer type. Preliminary drug predictions based on individual tumor characteristics support the use of PMed drug prescription in future comparative oncology studies.

## Discussion

In this study, our objective was to determine the feasibility of real time transcriptome analysis of canine tumors as part of a PMed strategy to allow selection of potentially active drugs for personalized patient therapy. The timeline from tumor biopsy to PMed report generation was <5 business days, confirming the practicality of prospective tumor collection, molecular profiling, and generation of an actionable PMed report in dogs with cancer. Tumor samples collected were of high quality measured by both histopathologic and molecular standards of nucleic acid integrity and yield.

MDS analysis revealed that canine tumor gene expression was strongly tied to cancer type. Although the number of histologic subgroups analyzed was small, the data was consistent. Additionally, clustergram analysis of personalized drug reports across samples demonstrated heterogeneity in predictions even within a single cancer type. This lends support to the inclusion of dogs with naturally occurring cancers in PMed preclinical studies, where patient-to-patient variability within a given cancer type (histology) exists. Indeed, review of the drug predictions derived from canine expression studies includes several therapeutic agents that are reasonably predicted to have efficacy in a given cancer (i.e. mitoxantrone in lymphoma) as well as agents not commonly used in that cancer but used in other cancer types (i.e. the knase inhibitor sunitinib in bladder cancer), and also drugs that are not commonly used in cancer patients (i.e. theophylline). This selection of PMed derived agents, supports the over-riding premise of this approach, since conventionally used drugs are included as options (proof of concept), but is extended by agents that may not be considered without this approach.

Breed and type-specific collections for Golden Retrievers with lymphoma, Scottish terriers with TCC, and an open histology/open breed cohort also allowed for comparisons across tumor type and breed. Cancer type-defined clusters trumped breed associations. However it is possible that, if sequenced, tumor mutational status might be more specifically descriptive of breed. Also of note, accrual was slower for some breed and cancer type-specific cohorts (notably American Cocker Spaniels with melanoma), and, therefore subsequent breed based efforts require additional large-scale incentivized accrual to be sustainable.

Companion animals with cancer have been increasingly used to provide insight into tumor biology and in clinical studies of drug development [15], [21], [22]. As noted above, this is particularly germane to PMed where traditional rodent xenograft models do not collectively represent the heterogeneity known to exist in a population of human patients with a given histological diagnosis of cancer [14], [23]. Comparative models may address challenges in the PMed field by providing both types of heterogeneity and as such the opportunity to ask if PMed guided interventions are associated with improved outcomes compared to conventional approaches. Furthermore, since PMed algorithms often define “first,” “second,” “third” tier agents, comparative oncology trials could test the clinical value of the first versus the second and third agents. Such agents may be offered as front line therapy for dogs with cancer alone or in combination with other cancer therapy. Comparative oncology trials could also allow the comparison of PMed algorithms (which are likely to be context-sensitive) through head-to-head trials to define the most successful approaches or scenarios for algorithm prescription[28]. With crossover rules for progressive disease, novel trial designs may also allow the evaluation of presumed “negative” (i.e. not predicted to be effective) agents compared to presumed “positive) agents (i.e. predicted to be effective). Further, components of the PMed approach may be individually tested and optimized through comparative models. Points of optimization may include defining the best sources of molecular data input, determining optimal biopsy collection techniques, evaluating informed algorithm generation, and exploring combinational therapy selection [26].

PMed comparative oncology studies are however limited by challenges in the translation of genomic signatures across species. For example, although there are data demonstrating similarities in gene expression between canine and human cancer types, the use of human expression signatures to query canine expression data is not well-established [24], [25] [27]. Further, in the case of gene expression data, the dearth of normal reference sets in comparative species can present a challenge. In this report the use of a canine normal tissue expression data set was utilized. Alternative options for such reference sets may include expression data from other tumor types. From the perspective that these analyses are aimed at identification of key deregulation phenomena, we expect that significant deregulation will be identified even when highly variable reference sets of tumors are used. All methods begin with basic z-scores and for genes that are deregulated these scores will be very significant regardless of the reference. In support of this, review of the drug selection data (Table S1) provides support for the validity of this cross-species approach. For example, the drug selection outcomes in canine lymphoma compared to other types disproportionately include cytotoxic drugs that are conventionally used to treat canine and human lymphoma. Similarly, the transitional cell carcinoma of the bladder disproportionately included several inhibitors of the cox-2 pathway. Interestingly these agents have been shown to be active and are under evaluation in canine and human bladder cancers.

Complex models are needed to effectively evaluate PMed study designs and this proof of concept trial validates the dog with cancer as a model for clinical evaluation of novel PMed approaches. It is now reasonable that dogs with cancer can begin to fill the gap in optimizing the delivery of these approaches for translation to human patients. Our study used operational, analytical and clinical aspects of a comparative approach to identify potentially active agents in spontaneously derived cancers. This study sets the foundation for trials that will become more integrative and comprehensive in nature though the generation and analysis of multiple dimensions of genomic data in conjunction with prospective clinical outcomes. Comparative oncology models have the potential to expedite this evaluation and lead advancements in personalized medicine.

## Methods

### Comparative Oncology Trials Consortium

The goals and infrastructure of the COTC have been previously described [14], [21], [29]. All COTC trial data were reported electronically and contemporaneously reviewed through the Cancer Central Clinical Database (C3D), a controlled-access database developed through the NCI's Center for Cancer Research (CCR) and Cancer Bioinformatics Grid (CaBIG) and modified for use in canine clinical trials [30].

### Study design and schedule

Client-owned pet dogs with histologically confirmed cancer, favorable performance status (grade 0 or 1 modified Eastern Cooperative Oncology Group (ECOG) performance status), and informed owner consent were eligible for enrollment. Specific subsets including Scottish terriers with transitional cell carcinoma of the bladder, golden retrievers with multi-centric lymphoma and all breeds with oral melanoma were eligible for enrollment. Eligibility criteria required a tumor amenable to a peripheral biopsy (except the cases with transitional cell carcinoma of the bladder). Only dogs with naïve disease were eligible for enrollment. Physical examination and laboratory [complete blood count (CBC), serum biochemical profile, urinalysis (UA)] evaluations were performed to evaluate eligibility prior to enrollment. Exclusion criteria removed dogs with significant co-morbidities (such as renal, liver, and heart failure or coagulopathy), history of chemotherapy (including corticosteroids in lymphoma cases and NSAIDs in TCC), radiation therapy or immunotherapy. All dogs were evaluated uniformly and treated within a defined clinical protocol with IACUC approval at each COTC enrollment site (Colorado State University, Michigan State University, Purdue University, Tufts University, University of Georgia, University of Minnesota, and University of Wisconsin-Madison). The NCI-Comparative Oncology Program (COP) reviewed the eligibility screening and approved trial entry of each patient. Potential adverse events related to the research protocol were monitored according to accepted VCOG-CTCAE criteria [18].

### Patient and Sample Tracking by Wiki

Defining the clinical turnaround time for prospective gene expression analysis and personalized medicine report generation was a main objective of this study. The Confluence Enterprise Wiki online database tracked the location of the specimens and time spent at each step of the QA/QC process. Confluence Enterprise Wiki was created by Atlassian and was utilized for this study. Researchers involved in the study were given usernames and passwords to access the common study site. At the start of the study 4 tables, one for each cohort, were constructed on the wiki space. When a patient enrolled, their patient ID, sex, date of birth, breed, and tumor type were entered into the table by the study monitor (CM). The specimens and results were tracked in real time with each investigator entering the date and time a sample arrived in their laboratory, when analysis began, and was completed, as well as the sample results. The wiki space allowed us to track the location of the specimens and derived data at each step of the QA/QC process.

### Sample Collections

#### Tumors

Tumor biopsies on Day 1 of the study were required from all dogs. Tumors must have been at least 3 cm in longest diameter to be eligible for biopsy. Biopsy techniques were prospectively defined by standard operating procedures (SOPs) and used uniformly at all participating COTC sites. Biopsies were collected by either a 10–14 gauge Tru-cut instrument, 6 mm punch biopsy, or an open biopsy technique. Biopsies for dogs with transitional cell carcinoma were collected by cystoscope or ultrasound guided. Two samples of at least 1 cm in length were obtained at various planes within the tumor to capture natural disease heterogeneity. The two planes were labeled as Tumor A and Tumor B. Each of the sections were bisected equally. Tumor A was divided and equal specimens placed in either RNAlater or formalin. Tumor B was divided and equal specimens placed in formalin or flash frozen. Tumor A samples (RNAlater) were shipped overnight on a −20 C ice pack to the Clinical Reference Laboratory (CRL) for RNA isolation. Tumor A and B samples (formalin) were shipped overnight on a −20 C ice pack to Colorado State University.

#### Plasma and serum

Plasma and serum samples were collected for all patients by standardized procedures on Day 1. These were flash frozen in liquid nitrogen and stored at −80 C. At the end of the study samples were shipped overnight on dry ice to the NCI-COP for permanent storage. These were stored for potential post hoc secondary analyses.

### Histopathology Review

Formalin-fixed, paraffin-embedded tumor biopsy samples were routinely sectioned, paraffin embedded, and stained with H&E for light microscopic evaluation. Histopathology quality assurance and control (QA/AC) assessment for all biopsies were performed by one pathologist (EJE). Data was subjectively classified into ordinal catagories: % tumor surface area was defined as the percentage surface area of each examined tissue that was determined to be tumor; % tumor nuclei was defined as the percentage of each examined tissue's nuclei that were determined to be tumor nuclei; % tumor necrosis was defined as the percentage of each examined tumor that was determined to be necrotic. Samples were evaluated for all 3 parameters (tumor surface, nuclei, and necrosis) at both the top and bottom of the tumor specimen and then averaged. Categories for percent tumor surface area were 0%–24%, 25%–49%, 50%–74%, and 75–100%. Categories for percent tumor nuclei were 0%–24%, 25%–49%, 50%–74%, and 75–100%. Categories for percent tumor necrosis were <10%, 10–20%, and >20%. Passing parameters were considered > or equal to 75% tumor and < or equal to 20% necrosis. Biopsies that failed to pass assessment were excluded from the remainder of the study. Representative microscopic images were captured for all examined samples and banked for future use.

### Total RNA Extraction

RNA isolation and QA/QC assessment from samples collected in the study was performed at a Clinical Laboratory Improvement Amendments (CLIA) accredited facility (Clinical Reference Laboratory, Lenexa, KS) to ensure quality laboratory testing. Standards defined here have been previously used for the conduct of human tissue processing and clinical trials (http://wwwn.cdc.gov/clia). RNA was extracted from canine tumor biopsy tissues (Tumor A) taken on Day 1 of the study and stored in RNA*later* stabilization solution (Ambion, Cat # AM7020). The quality measures for RNA isolation were quantity (total yield >20 ng) and integrity (A_260_/A_280_≥1.8, RIN≥8.0) measured by Nanodrop and Agilent Bioanalyzer respectively.

### Canine Genome 2.0 Expression Analysis

cDNA synthesis and amplification were accomplished using the NuGen Ovation Pico WTA System (Cat # 3300-12). Fifty nanograms of total RNA was used for cDNA synthesis using the following steps as per the Ovation Pico WTA System protocol: First Strand cDNA synthesis, second strand cDNA synthesis, cDNA purification, SPIA cDNA amplification, and amplified SPIA cDNA purification. The amplified DNA was checked for quality and quantity using the Nanodrop spectrophotometer. cDNA samples with a 260/280 ratio of ≥1.8 and a concentration above 5 µg in 30 ul were considered acceptable for further processing using the Affymetrix Canine genome 2.0 array.

Amplified cDNA samples generated with the NuGen Ovation Pico WTA system were used for fragmentation and labelling process using the NuGen Encore Biotin Module (Cat # 4200-12). The resulting fragmented and labeled cDNA was used for Affymetrix Canine 2.0 array hybridization. The hybridized arrays were washed and stained using GeneChip Hybridization, Wash and Stain Kit (Affymetrix, Cat # 900720).

Initial QC analysis of the scanned array was accomplished using the Affymetrix Expression Console Software. Background noise <100, % present call ≥30%, scale factor 100 and appropriate spike in control signals are necessary for adequate sample quality. Upon passing all criteria, MAS5.0 processed .CEL and normalized pivot .TXT files were extracted and deposited on a secure FTP site at Van Andel Research Institute (VARI) for subsequent analysis. This data has also been uploaded to GEO, accession #GSE51131.

### Bioinformatics

The general analytical workflow undertaken upon receipt of a tumor-derived gene expression profile is shown diagrammatically in **Figure 2** and is adapted from a workflow previously established in human neuroblastoma [19]. Gene expression data from each tumor was compared to a reference sample set in order to obtain a relative gene expression profile [20]. Each gene probeset was represented by a z-score depicting its expression in the tumor in terms of the number of standard-deviations from the mean expression in the reference set. The individual patient samples from the canine Affymetrix array probesets were converted to z-scores using a normal K-9 reference set based on the 39 samples in GEO data set GSE20113. In the cases where multiple probesets represented the same gene (Affymetrix canine 2.0 version 31 annotation) they were aggregated using the mean to a single value for the appropriate Entrez gene identifier. The canine Entrez gene identifiers were then converted to human Entrez ID's using the homolog data from the NCI database (ftp://ftp.ncbi.nih.gov/pub/HomoloGene/current/homologene.data dated 11/15/2010). Any canine ID's that had ambiguity in the mapping to human genes were removed and only values whose canine ID's exhibited clear and concise (one-to-one mapping from canine to human genes) conversion to human ID's were retained. The final step in the conversion process was to convert the human Entrez gene identifiers to the appropriate Affymetrix U133 Plus 2.0 probesets (U133 Plus 2.0 annotation version 31). Only concisely mapped Entrez gene IDs to Affymetrix probesets were retained. Use of the U133 Plus 2.0 probeset data facilitates the use of the standard workflow and application of the previously-detailed predictive methods developed for human subjects [19]. The standard workflow is capable of utilizing z-Score values associated with the U133 Plus 2.0 probesets. After this pre-processing step, data was submitted to the following collection of predictive methodologies to identify potential agents for consideration. All predictions are based on canine genomic data (tumor and normal tissues) but a human bioinformatics backbone as detailed:

#### Drug Target Expression

This first and most rudimentary method utilizes a human drug-target (mechanism of action) knowledge base and rules-based method to identify over-expressed genes (z-score ≥+3) in a patient's tumor that represent known molecular targets of antagonists, then match the appropriate drug from the knowledge base (example rule: IF EGFR Expression z-score ≥+3 THEN INDICATE Cetuximab). Multiple sources of public domain knowledge have been used to establish the internally-curated drug-target knowledge base including DrugBank, MetaCore (GeneGo-Thomson Reuters), MedTrack, PharmGKB, UpToDate and DrugDex (Thomson Reuters) [31], [32]. These rules are subject to change based on review of current literature evidence. Existing z-score thresholds of +3 or −3 were selected based on prior experience, but thresholds are variable by rule and can be adjusted as needed. The p values are derived from the z-score of the expression level used to trigger the rule – the greater the z-score, the lower the p value associated with the rule. 260 vetted drug-target rules covering 123 drugs across 260 unique targets were contained within the drug-target knowledge base used in this study (**Table S2**).

#### Biomarker Rules

Much like target expression, this method employs predefined and published rules maintained in a drug-biomarker database mined from public knowledge in which the efficacy of a specific drug has been associated with the expression of a specific molecular marker [9]. However, this method not only highlights drugs with predicted sensitivity, but also highlights drugs that may be contraindicated (insensitive drugs) on the basis of resistance rules (example rule: IF ERCC1 z-score ≥+3 THEN CONTRAINDICATE Oxaliplatin). Further, this method can take into account underexpressed genes (z-scores ≤−3). Currently, there are 34 biomarker rules indicating sensitivity to 14 FDA approved drugs in this database. Combining sensitive and resistance biomarker rules indicates 20 unique FDA approved drugs within the drug-biomarker rules knowledge base (**Table S3**).

#### Drug Response Signatures

This method reproduces the Connectivity Map (CMAP) concept initially developed by the Broad Institute in which the genomic consequence of drug exposure is used to connect drug effect to disease signatures [33]. The hypothesis underlying this method is that drugs that reverse the disease genotype (gene expression profile) towards normalcy have the potential to reverse the disease phenotype. The CMAP method is based on the exposure of four cell lines (MCF7, PC3, HL60, and SKMEL5) to a series of 142 small molecules and measurement of pre- and post-exposure gene expression profiles as described in the above reference. For our purposes, over- and under-expressed genes in the patient's tumor (z-scores ≥+2.0 or ≤−2.0 respectively) are compared to every array in the CMAP drug response signature database. Rank-based statistics are then used to identify drugs with a significant inverse connectivity to the disease genotype and to generate an enrichment score for each gene in the list. The drug list is a subset derived from CMAP and refined based on literature support (**Table S4**) and drug match scores are calculated using Kolmogorov-Smirnov statistics with p-values estimated using permutation testing with 50,000 permutations. Only those patterns that match with a p-value less than 0.05 are reported.

#### Drug Sensitivity Signatures

This method reproduces a previously published implementation of the Parametric Gene Set Enrichment Analysis (PGSEA) method using the NCI-60 cell line sensitivity data provided in the COMPARE database [34], [35], [36]. Gene expression signatures from the untreated NCI-60 lines associated with differential response to specific drugs on the basis of IC-50s from an *in vitro* drug screen reported in the COMPARE database are compared to the canine tumor-derived gene expression signature (all probesets from one sample standardized to z-score relative to the normal reference set) through the PGSEA analysis tool which in this iteration utilizes a one sample t-test to determine sensitivity to a subset of 11 drugs selected from the NCI-60 list (**Table S5**). Each drug is assigned a p-value for predictive efficacy and only those with a p-value less than 0.05 are reported. This approach is consistent with well-published methods for inferring drug sensitivity utilizing the NCI-60 cell line dataset [35], [36], [37].

#### Network Target Activity

This method predicts the activity (rather than expression) level of drug targets on the basis of a specific type of molecular network analysis referred to as topological analysis described previously [38]. This method utilizes gene expression data and pre-requisite knowledge of protein-protein interactions based on GeneGo topology analysis to predict upstream target activity on the basis of observed downstream transcriptional events selected from Affymetrix probes – either all z-scores ≥2 or the top 200 overexpressed probes (*K* in formula below). *K* thus represents a subset of a global interaction network of size *N*. Construction of a directed shortest path network connecting nodes from *K* to each other is performed utilizing the MetaCore GeneGO database of over 200,000 protein-protein and protein-small molecule interactions. The shortest path network, *S*, is constructed by building directed paths from each node in *K* to other nodes in *K*, traversing via other nodes in the global network as necessary. The number of node pairs in the shortest path network which are connected through each pair of nodes *i* and *j* is determined (*K_ij_*). This process is repeated in the global network to calculate the number of node pairs connected through nodes *i* and *j* (*N_ij_*). The probability that the number of nodes would be *K_ij_* or larger given the size of the input network and the distribution of shortest paths in the global network is given by:
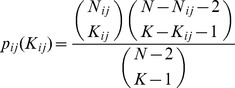
Hereby, the analysis output is the probability that a given drug target provides significant input to or output from a highly connected network identified in each tumor. Calculated p values are used to rank predictive scores and only those above 0.05 are reported. Currently, this method attempts to predict the activity of 260 unique drug targets spanning 123 FDA human approved drugs (**Table S2**).

### Personal Medicine Report Generation

Upon execution of the different analytical methodologies, a compiled report was generated. The personalized drug report conveys the predicted efficacy (or resistance in the case of biomarker resistance rules) of the drugs identified by each of the methods described above. These reports contain a summary section combining results of each method with scores that represent stronger indications for drugs that were predicted by multiple methods. These scores are adjusted for multiple methods and are based on the sum of scores from individual methods. In addition, each method contains scores reflecting the contribution of multiple genes to the suggested therapy. Further, personalized drug reports also associate public clinical and contextual knowledge to show any evidence that may support the use of the predicted drug in the context of the patient's disease state. The supporting evidence comes from a variety of sources including PubMed, clinicaltrials.gov and DrugDex (Thomson Reuters).

### Clinical Turnaround Time Monitoring

All sample processing time points were recorded on the COTC016 wiki page. This included the time of biopsy, time shipped from COTC site and time tumor specimens arrived at the Clinical Reference Laboratory (CRL) for expression analysis and Colorado State University (CSU) for histopathology analysis. Also included were the QA/QC analysis start and completion times at the CRL and CSU. Each lab entered the results in the wiki and uploaded data and/or representative images. All recorded times were listed in their respective time zone. When calculating the total elapsed time from biopsy shipment to PMed report generation, all times were converted to EST then military time for calculation purposes. Holiday and weekend hours were subtracted from total times to calculate business hours necessary for sample evaluation.

### Gene Expression Cluster Analysis

mRNA expression values for each gene in the canine tumor samples were normalized relative to the average expression of that gene in 40 canine normal tissue samples from a reference set [20]. The tumor to normal ratio and standard deviation in the tumor samples was used to calculate a z-score statistic for each gene (mRNA) as described above in Bioinformatics. mRNA z-scores and drug prediction scores for each sample were then used for separate multidimensional scaling (MDS) analyses for each data type. MDS coordinates were generated using the classical multidimensional scaling (cmdscale) function of the R statistical application (http://www.r-project.org; v2.14.1) based on sample to sample distances calculated using Pearson's correlation distance (one minus Pearson's correlation coefficient).

## Supporting Information

Figure S1 **Canine tumor gene expression signatures cluster independently of breed.** In a cursory evaluation of the potential effect of breed on tumor classification in this limited sample set, breed did not influence MDS analysis of gene expression z-scores. Both pure bred and mixed breed dog samples clustered by cancer type.
(TIF)

Table S1 **Summary of drug predictions: Top drug prediction by algorithm [−log(p) score] (Inferring gene(s) where applicable).** Drug recommendations based on the six drug prediction methodologies are shown for each tumor and includes the highest ranked recommended according to a summary score. This summary score gives more weight to drugs suggested by more than one algorithm.
(DOCX)

Table S2 **Expression and network-based drugs and targets.** Two hundred and sixty unique drug targets for 123 FDA approved human are shown alongside supporting evidence for the drug-target interactions that guideds4 inclusion in the network-based prediction algorithm.
(DOCX)

Table S3 **Biomarker rules.** Thirty-four biomarker rules are shown that match 20 FDA approved drugs to 22 targets according to sensitivity or resistance alongside supporting evidence for these interactions.
(DOCX)

Table S4 **Response signature drugs.** The subset of 107 CMAP drugs refined based on literature support and used to match drugs based on disease genotypes.
(DOCX)

Table S5 **Sensitivity profile drugs.** The subset of 11 drugs selected from the NCI-60 list COMPARE database and matched to gene expression signatures.
(DOCX)
