# Supplementary figures and images for: Prospective Molecular Profiling of Canine Cancers Provides a Clinically Relevant Comparative Model for Evaluating Personalized Medicine (PMed) Trials
Source: PLoS One. 2014 Mar 17;9(3):e90028. doi: 10.1371/journal.pone.0090028 (PMC3956546; doi:10.1371/journal.pone.0090028)

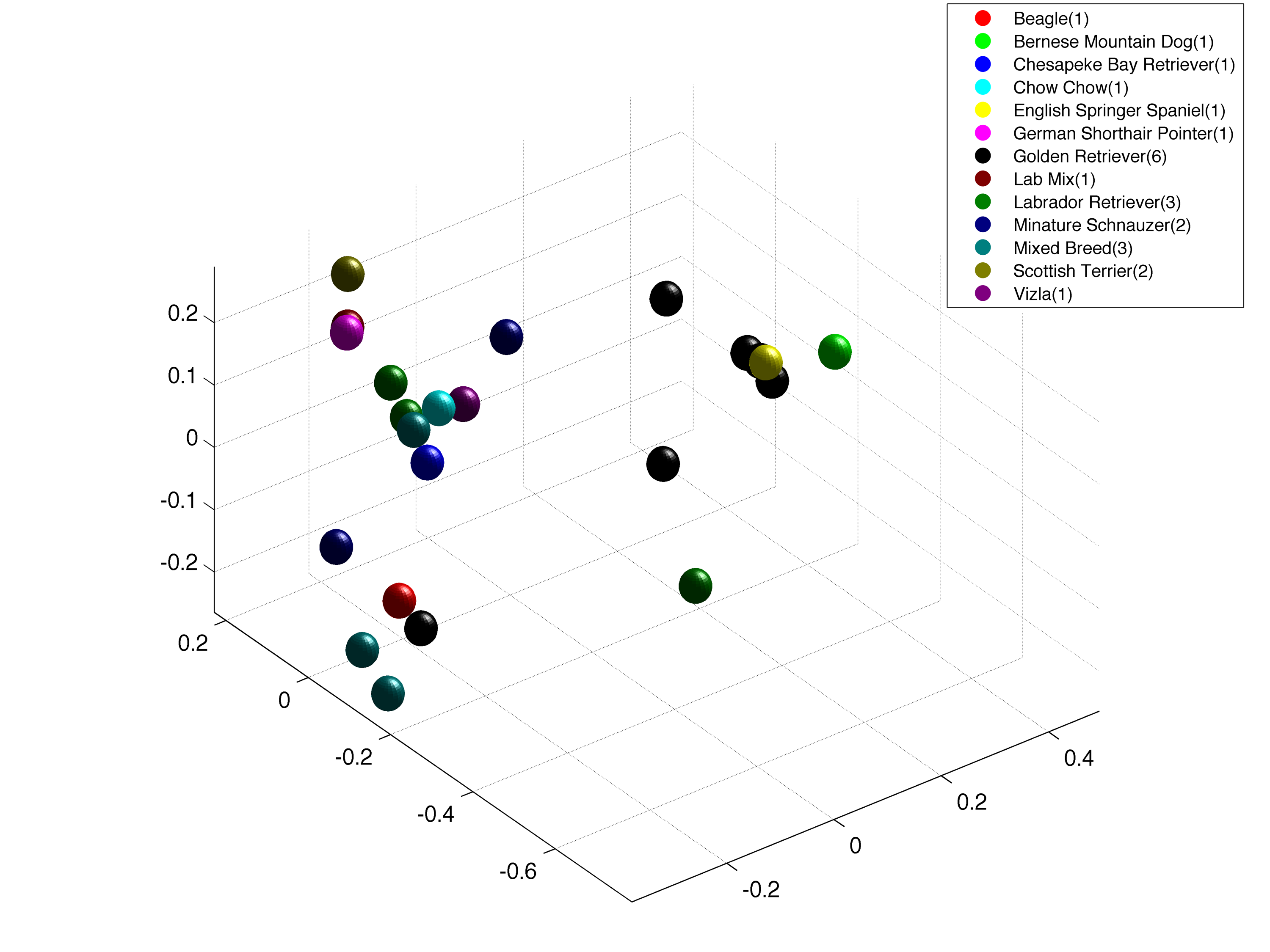

Supplement: Figure S1 — Canine tumor gene expression signatures cluster independently of breed. In a cursory evaluation of the potential effect of breed on tumor classification in this limited sample set, breed did not influence MDS analysis of gene expression z-scores. Both pure bred and mixed breed dog samples clustered by cancer type. (TIF) [file pone.0090028.s001.tif]
