# Supplementary material for: Prospective Molecular Profiling of Canine Cancers Provides a Clinically Relevant Comparative Model for Evaluating Personalized Medicine (PMed) Trials
Source: PLoS One. 2014 Mar 17;9(3):e90028. doi: 10.1371/journal.pone.0090028 (PMC3956546; doi:10.1371/journal.pone.0090028)
Supplement: Table S1 — Summary of drug predictions: Top drug prediction by algorithm [−log(p) score] (Inferring gene(s) where applicable). Drug recommendations based on the six drug prediction methodologies are shown for each tumor and includes the highest ranked recommended according to a summary score. This summary score gives more weight to drugs suggested by more than one algorithm. (DOCX) [file pone.0090028.s002.docx]

**Supplementary Table 1: Summary of Drug Predictions: Top Drug Prediction by Algorithm [-log(p) score] (Inferring Gene(s) where Applicable)**

| **Sample Id** | **Highest Ranked Drug Via Summary Score ^A^** | **Drug Target Expression^1^** | **Drug Response Signatures^2^** | **Drug Sensitivity Signatures^3^** | **Network Target Activity^4^** | **Biomarker Sensitivity Rules^5^** | **Biomarker Resistance Rules^6^** |
| --- | --- | --- | --- | --- | --- | --- | --- |
| **LYMPHOMAS** | | | | | | | |
| COTC016-0201 | vorinostat^1,4,5^ [18.90] | theophylline [42.4] (PDE4B) | irinotecan [2.54] | lomustine [5.92] | vorinostat [5.05,5.0] (HDAC3, HDAC2) | teniposide [18.6] (TOP2A) | capecitabine [5.83] (TYMS) fluorouracil [5.83] (TYMS) |
| COTC016-0209 | vorinostat^1,5^ [26.70] | sorafenib [172.00] (KIT) sunitinib [172.00] (KIT) imatinib [172.00] (KIT) | irinotecan [4.70] | daunorubicin [3.29] | plerixafor [3.25] (CXCR4) | gemcitabine [43.60] (DCK) | doxorubicin [10.90] (ABCC1) |
| COTC016-0502 | mitoxantrone^1,2^ [12.30] | thioguanine [25.60] (PPAT) | irinotecan [2.40] | lomustine [4.97] |  | topotecan [13.20] (TOP1) | capecitabine [4.36] (TYMS) fluorouracil [4.36] (TYMS) doxorubicin [4.33] (ABCC1) |
| COTC016-0504 | vorinostat^1,4,5^ [14.70] | mitoxantrone [60.10] (TOP2A) doxorubicin [60.10] (TOP2A) epirubicin [60.10] (TOP2A) etoposide [60.10] (TOP2A) | irinotecan [2.63] | lomustine [4.89] | vorinostat [4.37, 2.55] (HDAC2, HDAC1) | topotecan [67.40] (TOP1) |  |
| COTC016-0509 | trazodone^1^ [20.00] | trazodone [68.00, 18.20] (HTR2A) clozapine [68.00] (HTR2A) thioridazine [68.00] (HTR2A) | irinotecan [4.70] | carmustine [3.72] | axitinib [4.27, 3.49] (KDR, FLT1) sunitinib [4.27, 3.49] (KDR, FLT1) | bicalutamide [15.00] (AR) |  |
| COTC016-0510 | mitoxantrone^1,2^ [12.20] | theophylline [50.20] (PDE4B) | irinotecan [4.70] | lomustine [4.20] | thioridazine [1.94] (DRD1) | teniposide [24.10] (TOP2A) | capecitabine [5.96] (TYMS) fluorouracil [5.96] (TYMS) |
| COTC016-1304 | mitoxantrone^1,2^ [12.60] | mitoxantrone [46.60] (TOP2A) doxorubicin [46.60] (TOP2A) epirubicin [46.60] (TOP2A) etoposide [46.60] (TOP2A) | methotrexate [2.66] | carmustine [4.05] |  | teniposide [46.60] (TOP2A) | capecitabine [9.08] (TYMS) fluorouracil [9.08] (TYMS) |

| **TRANSITIONAL CELL CARCINOMAS** | | | | | | | |
| --- | --- | --- | --- | --- | --- | --- | --- |
| COTC016-0207 | sunitinib^1^ [20.00] | raloxifene [200.00] (ESR1) | methotrexate [3.01] | daunorubicin [3.77] | finasteride [3.43] (AR) | medroxyprogesterone [200.00] (ESR1) toremifene [200.00] (ESR1) | capecitabine [3.93] (TYMS) fluorouracil [3.93] (TYMS) |
| COTC016-0213 | sunitinib^1^ [20.00] | axitinib [54.90] (FLT1) sunitinib [54.90, 43.50] (FLT1, PDGFRA) | irinotecan [4.70] | carmustine [4.35] | decitabine [3.17] (DNMT1) | medroxyprogesterone [40.40] (ESR1) toremifene [40.40] (ESR1) |  |
| COTC016-0508 | sunitinib^1^ [20.00] | acetylsalicylic acid [229.00] (PTGS2) balsalazide [229.00] (PTGS2) bromfenac [229.00] (PTGS2) celecoxib [229.00] (PTGS2) diflunisal [229.00] (PTGS2) etodolac [229.00] (PTGS2) fenoprofen [229.00] (PTGS2) ibuprofen [229.00] (PTGS2) ketorolac [229.00] (PTGS2) lenalidomide [229.00] (PTGS2) meloxicam [229.00] (PTGS2) nabumetone [229.00] (PTGS2) tolmetin [229.00] (PTGS2) | irinotecan [4.70] methotrexate [4.70] | carmustine [3.55] | temsirolimus [3.54] (MTOR) | medroxyprogesterone [159.00] (ESR1) toremifene [159.00] (ESR1) | capecitabine [3.21] (TYMS) fluorouracil [3.21] (TYMS) |
| COTC016-1601 | sunitinib^1^ [20.00] | acetylsalicylic acid [108.00] (PTGS2) balsalazide [108.00] (PTGS2)  bromfenac [108.00] (PTGS2) celecoxib [108.00] (PTGS2) diflunisal [108.00] (PTGS2) etodolac [108.00] (PTGS2) fenoprofen [108.00] (PTGS2) ibuprofen [108.00] (PTGS2) ketorolac [108.00] (PTGS2) lenalidomide [108.00] (PTGS2) meloxicam [108.00] (PTGS2) nabumetone [108.00] (PTGS2) tolmetin [108.00] (PTGS2) | irinotecan [4.70] | carmustine [4.14] | donepezil [2.50] (ACHE) pyridostigmine [2.50] (ACHE) rivastigmine [2.50] (ACHE) tacrine [2.50] (ACHE) | bortezomib [22.30] (RAD23B) vorinostat [22.30] (RAD23B) | capecitabine [4.27] (TYMS) fluorouracil [4.27] (TYMS) |
| **OTHER CARCINOMAS** | | | | | | | |
| COTC016-0204 | vorinostat^4,5^ [13.00] | anakinra [100.00] (IL1R1) | irinotecan [2.96] | lomustine [2.28] | vorinostat [3.04] (HDAC2) | bortezomib [39.90] (RAD23B) vorinostat [39.90] (RAD23B) |  |
| COTC016-0501 | etoposide^1,2,3^ [13.00] | anakinra [129.00] (IL1R1) | irinotecan [4.70] | carmustine [4.35] |  | teniposide [18.50] (TOP2A) |  |

| **MELANOMAS** | | | | | | | |
| --- | --- | --- | --- | --- | --- | --- | --- |
| COTC016-0210 | temsirolimus^1^ [10.00] thioguanine^1^ [10.00] | temsirolimus [59.00] (MTOR) thioguanine [40.60] (PPAT) | irinotecan [4.70] | lomustine [4.89] | anakinra [2.91] (IL1R1) | topotecan [6.10] (TOP1) |  |
| COTC016-0211 | vorinostat^1,5^ [20.00] | thioguanine [25.50] (PPAT) | irinotecan [4.70] | carmustine [4.45] |  | bortezomib [25.60] (RAD23B) vorinostat [25.60] (RAD23B) |  |
| COTC016-0212 | vorinostat^1,4,5^ [13.40] | aminoglutethimide [29.00] (CYP19A1) anastrozole [29.00] (CYP19A1) exemestane [29.00] (CYP19A1) letrozole [29.00] (CYP19A1) | irinotecan [4.70] | carmustine [4.34] | vorinostat [2.72] (HDAC1) | teniposide [17.80] (TOP2A) | capecitabine [5.24] (TYMS) fluorouracil [5.24] (TYMS) |
| COTC016-0511 | vorinostat^4,5^ [17.20] | doxorubicin [18.30] (TOP2A) etoposide [18.30] (TOP2A) mitoxantrone [18.30] (TOP2A) epirubicin [18.30] (TOP2A) | irinotecan [2.37] | carmustine [4.45] | vorinostat [3.62] (HDAC1) | teniposide [18.30] (TOP2A) |  |
| COTC016-1502 | vorinostat^1,5^ [16.30] | acetylsalicylic acid [100.00] (PTGS2) balsalazide [100.00] (PTGS2)  bromfenac [100.00] (PTGS2) celecoxib [100.00] (PTGS2) diflunisal [100.00] (PTGS2) etodolac [100.00] (PTGS2) fenoprofen [100.00] (PTGS2) ibuprofen [100.00] (PTGS2) ketorolac [100.00] (PTGS2) lenalidomide [100.00] (PTGS2) meloxicam [100.00] (PTGS2) nabumetone [100.00] (PTGS2) tolmetin [100.00] (PTGS2) | irinotecan [4.70] | daunorubicin [3.88] | anakinra [2.99] (IL1R1) | bortezomib [22.10] (RAD23B) vorinostat [22.10] (RAD23B) | doxorubicin [4.43] (ABCC1) |
| **SARCOMAS** | | | | | | | |
| COTC016-0202 | vorinostat^1,5^ [13.50] | felodipine [10.30] (CACNA2D1) nifedipine [10.30] (CACNA2D1) | irinotecan [4.70] | daunorubicin [4.53] | anakinra [2.31] (IL1R1) | bortezomib [14.10] (RAD23B) vorinostat [14.10] (RAD23B) |  |
| COTC016-0203 | abciximab^1^ [10.00] bortezomib^1^ [10.00] vorinostat^1^ [10.00] | abciximab [13.10] (ITGB3) | methotrexate [4.10] | carmustine 4.20] | chlorpromazine [2.32] (DRD2) clozapine [2.32] (DRD2) prochlorperazine [2.32] (DRD2) | bortezomib [14.80] (RAD23B) vorinostat [14.80] (RAD23B) |  |
| COTC016-0205 | sunitinib^1^ [22.80] | anakinra [120.00] (IL1R1) | irinotecan [4.70] | lomustine [4.05] |  | bortezomib [7.94] (RAD23B) vorinostat [7.94] (RAD23B) |  |
| COTC016-0206 | sunitinib^1^ [29.90] | anakinra [100.00] (IL1R1) | irinotecan [4.70] | carmustine [3.83] | abciximab [3.07] (ITGB3) | bortezomib [32.10] (RAD23B) vorinostat [32.10] (RAD23B) | capecitabine [4.07] (TYMS) fluorouracil [4.07] (TYMS) |
| COTC016-0505 | sunitinib^1^ [43.30] | axitinib [100.00] (FLT1) sunitinib [100.00, 41.30, 13.80, 13.20, 3.35] (FLT1, KIT, PDGFRA, FLT4, KDR) | irinotecan [4.70] | carmustine [3.79] | drotrecogin alfa [2.99] (SERPINE1) | topotecan [3.54] (TOP1) |  |
|  |  |  |  |  |  |  |  |
| COTC016-1303 | thioguanine^1,3^ [10.70] | raloxifene [152.00] (ESR1) | methotrexate [3.12] | carmustine [3.79] |  | medroxyprogesterone [152.00] (ESR1) toremifene [152.00] (ESR1) | capecitabine [4.42] (TYMS) fluorouracil [4.42] (TYMS) |

^a^ Individual methods predicting efficacy for the identified drug are denoted 1-5. The Summary score represents a combined score of all methods where individual methods and biomarker rules are capped at a maximum -log(p) value of 10.0.
