# Supplementary material for: Prospective Molecular Profiling of Canine Cancers Provides a Clinically Relevant Comparative Model for Evaluating Personalized Medicine (PMed) Trials
Source: PLoS One. 2014 Mar 17;9(3):e90028. doi: 10.1371/journal.pone.0090028 (PMC3956546; doi:10.1371/journal.pone.0090028)
Supplement: Table S2 — Expression and network-based drugs and targets. Two hundred and sixty unique drug targets for 123 FDA approved human are shown alongside supporting evidence for the drug-target interactions that guideds4 inclusion in the network-based prediction algorithm. (DOCX) [file pone.0090028.s003.docx]

**Supplementary Table 2: Expression and Network-Based Drugs and Targets**

| **Generic Drug Name** | **Drug Target** | **Entrez ID** | **Evidence of Interaction (Internal and Specific)** |
| --- | --- | --- | --- |
| abciximab | ITGB3 | 3690 | http://portal.genego.com/cgi/regulation/link_info.cgi?id=-73223303 |
| acarbose | AMY2A | 279 | http://portal.genego.com/cgi/regulation/link_info.cgi?id=40475 |
| acarbose | GAA | 2548 | http://portal.genego.com/cgi/regulation/link_info.cgi?id=40476 |
| acetylsalicylic acid | PTGS1 | 5742 | http://portal.genego.com/cgi/regulation/link_info.cgi?id=1187 |
| acetylsalicylic acid | PTGS2 | 5743 | http://portal.genego.com/cgi/regulation/link_info.cgi?id=771 |
| adalimumab | TNF | 7124 | http://portal.genego.com/cgi/regulation/link_info.cgi?id=-593193555 |
| amiloride | SCNN1A | 6337 | http://portal.genego.com/cgi/regulation/link_info.cgi?id=-886638296 |
| amiloride | SCNN1B | 6338 | http://portal.genego.com/cgi/regulation/link_info.cgi?id=-2056673745 |
| amiloride | SCNN1G | 6340 | http://portal.genego.com/cgi/regulation/link_info.cgi?id=-866679838 |
| aminoglutethimide | CYP19A1 | 1588 | http://portal.genego.com/cgi/regulation/link_info.cgi?id=36736 |
| anakinra | IL1R1 | 3554 | http://portal.genego.com/cgi/regulation/link_info.cgi?id=-574552034 |
| anastrozole | CYP19A1 | 1588 | http://portal.genego.com/cgi/regulation/link_info.cgi?id=36733 |
| argatroban | F2 | 2147 | http://portal.genego.com/cgi/regulation/link_info.cgi?id=-1985222470 |
| atorvastatin | HMGCR | 3156 | http://portal.genego.com/cgi/regulation/link_info.cgi?id=23777 |
| azacitidine | DNMT1 | 1786 | http://portal.genego.com/cgi/entity_page.cgi?id=3020&term=100 |
| balsalazide | PTGS1 | 5742 | http://portal.genego.com/cgi/regulation/link_info.cgi?id=-1159887253 |
| balsalazide | PTGS2 | 5743 | http://portal.genego.com/cgi/regulation/link_info.cgi?id=-914401336 |
| bevacizumab | VEGFA | 7422 | http://portal.genego.com/cgi/regulation/link_info.cgi?id=-427826084 |
| bicalutamide | AR | 367 | http://portal.genego.com/cgi/regulation/link_info.cgi?id=41472 |
| bivalirudin | F2 | 2147 | http://portal.genego.com/cgi/regulation/link_info.cgi?id=-1677271829 |
| bortezomib | AKT1 | 207 | http://portal.genego.com/cgi/regulation/link_info.cgi?id=-1126348344 |
| bortezomib | NFKB1 | 4790 | http://portal.genego.com/cgi/regulation/link_info.cgi?id=-24668637 |
| bortezomib | NFKB2 | 4791 | http://portal.genego.com/cgi/regulation/link_info.cgi?id=-24668637 |
| bromfenac | PTGS1 | 5742 | http://portal.genego.com/cgi/regulation/link_info.cgi?id=-1928249663 |
| bromfenac | PTGS2 | 5743 | http://portal.genego.com/cgi/regulation/link_info.cgi?id=-207652417 |
| caffeine | ADORA1 | 134 | http://portal.genego.com/cgi/regulation/link_info.cgi?id=-446183328 |
| caffeine | ADORA2A | 135 | http://portal.genego.com/cgi/regulation/link_info.cgi?id=-644030522 |
| caffeine | ADORA2B | 136 | http://portal.genego.com/cgi/regulation/link_info.cgi?id=783 |
| carbamazepine | SCN5A | 6331 | http://portal.genego.com/cgi/regulation/link_info.cgi?id=-174977738 |
| carbidopa | DDC | 1644 | http://portal.genego.com/cgi/regulation/link_info.cgi?id=35809 |
| celecoxib | PDPK1 | 5170 | http://portal.genego.com/cgi/regulation/link_info.cgi?id=-813966096 |
| celecoxib | PTGS2 | 5743 | http://portal.genego.com/cgi/regulation/link_info.cgi?id=35733 |
| cetuximab | EGFR | 1956 | http://portal.genego.com/cgi/regulation/link_info.cgi?id=-974239227 |
| chlorpromazine | DRD1 | 1812 | http://portal.genego.com/cgi/regulation/link_info.cgi?id=-1640345421 |
| chlorpromazine | DRD2 | 1813 | http://portal.genego.com/cgi/regulation/link_info.cgi?id=36823 |
| chlorpromazine | HTR6 | 3362 | http://portal.genego.com/cgi/regulation/link_info.cgi?id=-367468966 |
| chlorpromazine | HTR7 | 3363 | http://portal.genego.com/cgi/regulation/link_info.cgi?id=-2018603150 |
| clofarabine | POLA1 | 5422 | http://portal.genego.com/cgi/regulation/link_info.cgi?id=-386702412 |
| clofarabine | RRM1 | 6240 | http://portal.genego.com/cgi/regulation/link_info.cgi?id=-228942783 |
| clopidogrel | P2RY12 | 64805 | http://portal.genego.com/cgi/regulation/link_info.cgi?id=-2017118818 |
| clozapine | CHRM1 | 1128 | http://portal.genego.com/cgi/regulation/link_info.cgi?id=37685 |
| clozapine | DRD2 | 1813 | http://portal.genego.com/cgi/regulation/link_info.cgi?id=38896 |
| clozapine | DRD3 | 1814 | http://portal.genego.com/cgi/regulation/link_info.cgi?id=-1697874080 |
| clozapine | DRD4 | 1815 | http://portal.genego.com/cgi/regulation/link_info.cgi?id=50797 |
| clozapine | HTR2A | 3356 | http://portal.genego.com/cgi/regulation/link_info.cgi?id=-247727960 |
| colchicine | TUBB2A | 7280 | http://portal.genego.com/cgi/regulation/link_info.cgi?id=-1421316874 |
| colchicine | TUBB | 203068 | http://portal.genego.com/cgi/regulation/link_info.cgi?id=-1421316874 |
| colchicine | TUBB2B | 347733 | http://portal.genego.com/cgi/regulation/link_info.cgi?id=-1421316874 |
| cyclosporin | PPP3CA | 5530 | http://portal.genego.com/cgi/regulation/link_info.cgi?id=-148446501 |
| cyclosporin | PPP3CB | 5532 | http://portal.genego.com/cgi/regulation/link_info.cgi?id=-393250108 |
| cyclosporin | PPP3CC | 5533 | http://portal.genego.com/cgi/regulation/link_info.cgi?id=14970 |
| cyclosporin | PPP3R1 | 5534 | http://portal.genego.com/cgi/regulation/link_info.cgi?id=-847696385 |
| cyclosporin | PPP3R2 | 5535 | http://portal.genego.com/cgi/regulation/link_info.cgi?id=-1609107488 |
| dantrolene | RYR1 | 6261 | http://portal.genego.com/cgi/regulation/link_info.cgi?id=-1281771032 |
| dasatinib | ABL1 | 25 | http://portal.genego.com/cgi/regulation/link_info.cgi?id=-1024740657 |
| dasatinib | EPHA2 | 1969 | http://portal.genego.com/cgi/regulation/link_info.cgi?id=-1243416236 |
| dasatinib | KIT | 3815 | http://portal.genego.com/cgi/regulation/link_info.cgi?id=-2009868571 |
| dasatinib | SRC | 6714 | http://portal.genego.com/cgi/regulation/link_info.cgi?id=-1988095900 |
| daunorubicin | ABCB1 | 5243 | http://portal.genego.com/cgi/regulation/link_info.cgi?id=-1359070108 |
| daunorubicin | TOP2A | 7153 | http://portal.genego.com/cgi/regulation/link_info.cgi?id=-234295135 |
| daunorubicin | TOP2B | 7155 | http://portal.genego.com/cgi/regulation/link_info.cgi?id=-234295135 |
| decitabine | DNMT1 | 1786 | http://portal.genego.com/cgi/regulation/link_info.cgi?id=-308241261 |
| denileukin diftitox | IL2RA | 3559 | http://portal.genego.com/cgi/regulation/link_info.cgi?id=-2089094660 |
| denileukin diftitox | IL2RB | 3560 | http://portal.genego.com/cgi/regulation/link_info.cgi?id=-2089094660 |
| denileukin diftitox | IL2RG | 3561 | http://portal.genego.com/cgi/regulation/link_info.cgi?id=-2089094660 |
| dexamethasone | IL6 | 3569 | http://portal.genego.com/cgi/regulation/link_info.cgi?id=-1110855848 |
| diflunisal | PTGS1 | 5742 | http://portal.genego.com/cgi/regulation/link_info.cgi?id=-1925644482 |
| diflunisal | PTGS2 | 5743 | http://portal.genego.com/cgi/regulation/link_info.cgi?id=-1034178617 |
| donepezil | ACHE | 43 | http://portal.genego.com/cgi/regulation/link_info.cgi?id=37644 |
| dorzolamide | CA2 | 760 | http://portal.genego.com/cgi/regulation/link_info.cgi?id=-1902457439 |
| doxorubicin | ABCB1 | 5243 | http://portal.genego.com/cgi/regulation/link_info.cgi?id=-1717872266 |
| doxorubicin | TOP2A | 7153 | http://portal.genego.com/cgi/regulation/link_info.cgi?id=-396457635 |
| doxorubicin | TOP2B | 7155 | http://portal.genego.com/cgi/regulation/link_info.cgi?id=-396457635 |
| doxycycline | IL1A | 3552 | http://portal.genego.com/cgi/regulation/link_info.cgi?id=-1743732904 |
| doxycycline | IL1B | 3553 | http://portal.genego.com/cgi/regulation/link_info.cgi?id=49075 |
| doxycycline | MMP1 | 4312 | http://portal.genego.com/cgi/regulation/link_info.cgi?id=-95394198 |
| doxycycline | MMP3 | 4314 | http://portal.genego.com/cgi/regulation/link_info.cgi?id=-1133483957 |
| doxycycline | MMP9 | 4318 | http://portal.genego.com/cgi/regulation/link_info.cgi?id=-1217473030 |
| doxycycline | MMP13 | 4322 | http://portal.genego.com/cgi/regulation/link_info.cgi?id=17851 |
| doxycycline | TNF | 7124 | http://portal.genego.com/cgi/regulation/link_info.cgi?id=-486438642 |
| drotrecogin alfa | F5 | 2153 | http://portal.genego.com/cgi/regulation/link_info.cgi?id=-2077581020 |
| drotrecogin alfa | F8 | 2157 | http://portal.genego.com/cgi/regulation/link_info.cgi?id=-75133205 |
| drotrecogin alfa | SERPINE1 | 5054 | http://portal.genego.com/cgi/regulation/link_info.cgi?id=-1973947292 |
| dutasteride | SRD5A1 | 6715 | http://portal.genego.com/cgi/regulation/link_info.cgi?id=-289591183 |
| dutasteride | SRD5A2 | 6716 | http://portal.genego.com/cgi/regulation/link_info.cgi?id=-1177623291 |
| epirubicin | TOP2A | 7153 | http://portal.genego.com/cgi/regulation/link_info.cgi?id=-1841398517 |
| epirubicin | TOP2B | 7155 | http://portal.genego.com/cgi/regulation/link_info.cgi?id=-1808390879 |
| erlotinib | EGFR | 1956 | http://portal.genego.com/cgi/regulation/link_info.cgi?id=-928952182 |
| estradiol | ETS1 | 2113 | http://portal.genego.com/cgi/regulation/link_info.cgi?id=-49681977 |
| etodolac | PTGS1 | 5742 | http://portal.genego.com/cgi/regulation/link_info.cgi?id=44444 |
| etodolac | PTGS2 | 5743 | http://portal.genego.com/cgi/regulation/link_info.cgi?id=44434 |
| etoposide | TOP2A | 7153 | http://portal.genego.com/cgi/regulation/link_info.cgi?id=-1368984564 |
| etoposide | TOP2B | 7155 | http://portal.genego.com/cgi/regulation/link_info.cgi?id=-1715276422 |
| exemestane | CYP19A1 | 1588 | http://portal.genego.com/cgi/regulation/link_info.cgi?id=36729 |
| felodipine | CACNA2D1 | 781 | http://portal.genego.com/cgi/regulation/link_info.cgi?id=-32617604 |
| fenoprofen | PTGS1 | 5742 | http://portal.genego.com/cgi/regulation/link_info.cgi?id=46411 |
| fenoprofen | PTGS2 | 5743 | http://portal.genego.com/cgi/regulation/link_info.cgi?id=46412 |
| finasteride | AR | 367 | http://portal.genego.com/cgi/regulation/link_info.cgi?id=-317197736 |
| finasteride | SRD5A1 | 6715 | http://portal.genego.com/cgi/regulation/link_info.cgi?id=-2070128870 |
| finasteride | SRD5A2 | 6716 | http://portal.genego.com/cgi/regulation/link_info.cgi?id=52147 |
| fluphenazine | HTR1B | 3351 | http://portal.genego.com/cgi/regulation/link_info.cgi?id=-2051208523 |
| flutamide | AR | 367 | http://portal.genego.com/cgi/regulation/link_info.cgi?id=-1534998264 |
| flutamide | PEG10 | 23089 | http://portal.genego.com/cgi/regulation/link_info.cgi?id=-1233446938 |
| fulvestrant | AR | 367 | http://portal.genego.com/cgi/regulation/link_info.cgi?id=-5158445 |
| fulvestrant | ESR2 | 2100 | http://portal.genego.com/cgi/regulation/link_info.cgi?id=34436 |
| gefitinib | EGFR | 1956 | http://portal.genego.com/cgi/regulation/link_info.cgi?id=-201452800 |
| gefitinib | ERBB2 | 2064 | http://portal.genego.com/cgi/regulation/link_info.cgi?id=-1372265794 |
| hydroxyurea | RRM1 | 6240 | http://portal.genego.com/cgi/regulation/link_info.cgi?id=-794396963 |
| ibuprofen | PTGS1 | 5742 | http://portal.genego.com/cgi/regulation/link_info.cgi?id=43745 |
| ibuprofen | PTGS2 | 5743 | http://portal.genego.com/cgi/regulation/link_info.cgi?id=43744 |
| imatinib | ABL1 | 25 | http://portal.genego.com/cgi/regulation/link_info.cgi?id=-1617151951 |
| imatinib | KIT | 3815 | http://portal.genego.com/cgi/regulation/link_info.cgi?id=-85922532 |
| imatinib | PDGFRA | 5156 | http://portal.genego.com/cgi/regulation/link_info.cgi?id=-1912204591 |
| imatinib | PDGFRB | 5159 | http://portal.genego.com/cgi/regulation/link_info.cgi?id=-1912204591 |
| imatinib | PDGFC | 56034 | http://portal.genego.com/cgi/regulation/link_info.cgi?id=-29574362 |
| irinotecan | TOP1 | 7150 | http://portal.genego.com/cgi/regulation/link_info.cgi?id=-65764914 |
| ketorolac | PTGS1 | 5742 | http://portal.genego.com/cgi/regulation/link_info.cgi?id=44206 |
| ketorolac | PTGS2 | 5743 | http://portal.genego.com/cgi/regulation/link_info.cgi?id=44205 |
| lansoprazole | ATP4A | 495 | http://portal.genego.com/cgi/regulation/link_info.cgi?id=-983707652 |
| lapatinib | EGFR | 1956 | http://portal.genego.com/cgi/regulation/link_info.cgi?id=-199193427 |
| lapatinib | ERBB2 | 2064 | http://portal.genego.com/cgi/regulation/link_info.cgi?id=-2066150089 |
| leflunomide | ALOX5 | 240 | http://portal.genego.com/cgi/regulation/link_info.cgi?id=-1930995799 |
| leflunomide | DHODH | 1723 | http://portal.genego.com/cgi/regulation/link_info.cgi?id=-1023567281 |
| leflunomide | PDGFRB | 5159 | http://portal.genego.com/cgi/regulation/link_info.cgi?id=-925073040 |
| leflunomide | PTGS2 | 5743 | http://portal.genego.com/cgi/regulation/link_info.cgi?id=-1825952241 |
| lenalidomide | PTGS2 | 5743 | http://portal.genego.com/cgi/regulation/link_info.cgi?id=-1976707877 |
| lenalidomide | TNF | 7124 | http://portal.genego.com/cgi/regulation/link_info.cgi?id=-11756618 |
| lenalidomide | VEGFA | 7422 | http://portal.genego.com/cgi/regulation/link_info.cgi?id=-1697094719 |
| lepirudin | F2 | 2147 | http://portal.genego.com/cgi/regulation/link_info.cgi?id=-264314631 |
| letrozole | CYP19A1 | 1588 | http://portal.genego.com/cgi/regulation/link_info.cgi?id=36734 |
| lovastatin | HMGCR | 3156 | http://portal.genego.com/cgi/regulation/link_info.cgi?id=-1338011689 |
| megestrol | ESR1 | 2099 | http://portal.genego.com/cgi/regulation/link_info.cgi?id=-540758820 |
| meloxicam | PTGS1 | 5742 | http://portal.genego.com/cgi/regulation/link_info.cgi?id=45600 |
| meloxicam | PTGS2 | 5743 | http://portal.genego.com/cgi/regulation/link_info.cgi?id=44433 |
| methimazole | TPO | 7173 | http://portal.genego.com/cgi/regulation/link_info.cgi?id=-36410280 |
| methotrexate | DHFR | 1719 | http://portal.genego.com/cgi/regulation/link_info.cgi?id=-2002914155 |
| minocycline | MMP9 | 4318 | http://portal.genego.com/cgi/regulation/link_info.cgi?id=-1311156137 |
| mitoxantrone | TOP2A | 7153 | http://portal.genego.com/cgi/regulation/link_info.cgi?id=-703537190 |
| nabumetone | PTGS1 | 5742 | http://portal.genego.com/cgi/regulation/link_info.cgi?id=-1505071720 |
| nabumetone | PTGS2 | 5743 | http://portal.genego.com/cgi/regulation/link_info.cgi?id=-2097946843 |
| nifedipine | CACNA2D1 | 781 | http://portal.genego.com/cgi/regulation/link_info.cgi?id=-1733922288 |
| nitisinone | HPD | 3242 | http://portal.genego.com/cgi/regulation/link_info.cgi?id=36473 |
| octreotide | SSTR1 | 6751 | http://portal.genego.com/cgi/regulation/link_info.cgi?id=-1944565164 |
| octreotide | SSTR2 | 6752 | http://portal.genego.com/cgi/regulation/link_info.cgi?id=18867 |
| octreotide | SSTR3 | 6753 | http://portal.genego.com/cgi/regulation/link_info.cgi?id=-1504844108 |
| octreotide | SSTR4 | 6754 | http://portal.genego.com/cgi/regulation/link_info.cgi?id=-892364965 |
| octreotide | SSTR5 | 6755 | http://portal.genego.com/cgi/regulation/link_info.cgi?id=-837757382 |
| omeprazole | ATP4A | 495 | http://portal.genego.com/cgi/regulation/link_info.cgi?id=-614383575 |
| orlistat | PNLIP | 5406 | http://portal.genego.com/cgi/regulation/link_info.cgi?id=17135 |
| oxaliplatin | BCL2 | 596 | http://portal.genego.com/cgi/regulation/link_info.cgi?id=-1445120677 |
| oxaliplatin | BCL2L1 | 598 | http://portal.genego.com/cgi/regulation/link_info.cgi?id=-1641107820 |
| oxaprozin | PTGS1 | 5742 | http://portal.genego.com/cgi/regulation/link_info.cgi?id=44442 |
| paclitaxel | TUBB2A | 7280 | http://portal.genego.com/cgi/regulation/link_info.cgi?id=-764679415 |
| paclitaxel | TUBB | 203068 | http://portal.genego.com/cgi/regulation/link_info.cgi?id=-764679415 |
| paclitaxel | TUBB2B | 347733 | http://portal.genego.com/cgi/regulation/link_info.cgi?id=-764679415 |
| paclitaxel albumin-bound | TUBB2A | 7280 | http://portal.genego.com/cgi/regulation/link_info.cgi?id=-764679415 |
| paclitaxel albumin-bound | TUBB | 203068 | http://portal.genego.com/cgi/regulation/link_info.cgi?id=-764679415 |
| paclitaxel albumin-bound | TUBB2B | 347733 | http://portal.genego.com/cgi/regulation/link_info.cgi?id=-764679415 |
| panitumumab | EGFR | 1956 | http://portal.genego.com/cgi/regulation/link_info.cgi?id=-509115237 |
| pemetrexed | ATIC | 471 | http://portal.genego.com/cgi/regulation/link_info.cgi?id=-795191780 |
| pemetrexed | DHFR | 1719 | http://portal.genego.com/cgi/regulation/link_info.cgi?id=-1480187824 |
| pemetrexed | GART | 2618 | http://portal.genego.com/cgi/regulation/link_info.cgi?id=25544 |
| phenelzine | MAOA | 4128 | http://portal.genego.com/cgi/regulation/link_info.cgi?id=49775 |
| phentolamine | ADRA2A | 150 | http://portal.genego.com/cgi/regulation/link_info.cgi?id=-1866862979 |
| piroxicam | PTGS2 | 5741 | http://portal.genego.com/cgi/regulation/link_info.cgi?id=-1620721358 |
| piroxicam | PTGS1 | 5742 | http://portal.genego.com/cgi/regulation/link_info.cgi?id=35740 |
| plerixafor | CXCR4 | 7852 | http://portal.genego.com/cgi/regulation/link_info.cgi?id=-702217872 |
| pravastatin | HMGCR | 3156 | http://portal.genego.com/cgi/regulation/link_info.cgi?id=-148012302 |
| pravastatin | MMP2 | 4313 | http://portal.genego.com/cgi/regulation/link_info.cgi?id=-1385649724 |
| pravastatin | MMP9 | 4318 | http://portal.genego.com/cgi/regulation/link_info.cgi?id=-1215410816 |
| pravastatin | MMP14 | 4323 | http://portal.genego.com/cgi/regulation/link_info.cgi?id=-2078580110 |
| pravastatin | TIMP2 | 7077 | http://portal.genego.com/cgi/regulation/link_info.cgi?id=-2081204048 |
| prochlorperazine | DRD2 | 1813 | http://portal.genego.com/cgi/regulation/link_info.cgi?id=-803226380 |
| pyridostigmine | ACHE | 43 | http://portal.genego.com/cgi/regulation/link_info.cgi?id=44018 |
| rabeprazole | ATP4B | 496 | http://portal.genego.com/cgi/regulation/link_info.cgi?id=-1098123581 |
| raloxifene | ESR1 | 2099 | http://portal.genego.com/cgi/regulation/link_info.cgi?id=34437 |
| raloxifene | ESR2 | 2100 | http://portal.genego.com/cgi/regulation/link_info.cgi?id=3183 |
| ramipril | ACE | 1636 | http://portal.genego.com/cgi/regulation/link_info.cgi?id=-215451934 |
| rituximab | MS4A1 | 931 | http://portal.genego.com/cgi/regulation/link_info.cgi?id=-1293420705 |
| rivastigmine | ACHE | 43 | http://portal.genego.com/cgi/regulation/link_info.cgi?id=38302 |
| rivastigmine | BCHE | 590 | http://portal.genego.com/cgi/regulation/link_info.cgi?id=38303 |
| sildenafil | PDE5A | 8654 | http://portal.genego.com/cgi/regulation/link_info.cgi?id=27312 |
| simvastatin | RHOA | 387 | http://portal.genego.com/cgi/regulation/link_info.cgi?id=-72413115 |
| simvastatin | HMGCR | 3156 | http://portal.genego.com/cgi/regulation/link_info.cgi?id=-525730519 |
| simvastatin | IGF1 | 3479 | http://portal.genego.com/cgi/regulation/link_info.cgi?id=-1354112715 |
| sirolimus | FKBP1A | 2280 | http://portal.genego.com/cgi/regulation/link_info.cgi?id=3326 |
| sirolimus | MTOR | 2475 | http://portal.genego.com/cgi/regulation/link_info.cgi?id=8254 |
| sorafenib | BRAF | 673 | http://portal.genego.com/cgi/regulation/link_info.cgi?id=-1668882218 |
| sorafenib | FLT1 | 2321 | http://portal.genego.com/cgi/regulation/link_info.cgi?id=-1313030443 |
| sorafenib | FLT3 | 2322 | http://portal.genego.com/cgi/regulation/link_info.cgi?id=-1788573953 |
| sorafenib | FLT4 | 2324 | http://portal.genego.com/cgi/regulation/link_info.cgi?id=-2061144740 |
| sorafenib | KDR | 3791 | http://portal.genego.com/cgi/regulation/link_info.cgi?id=-219392052 |
| sorafenib | KIT | 3815 | http://portal.genego.com/cgi/regulation/link_info.cgi?id=-600715072 |
| sorafenib | PDGFRB | 5159 | http://portal.genego.com/cgi/regulation/link_info.cgi?id=-483381446 |
| sorafenib | RAF1 | 5894 | http://portal.genego.com/cgi/regulation/link_info.cgi?id=-848339586 |
| sorafenib | RET | 5979 | http://portal.genego.com/cgi/regulation/link_info.cgi?id=-36254124 |
| sunitinib | CSF1R | 1436 | http://portal.genego.com/cgi/regulation/link_info.cgi?id=-794065103 |
| sunitinib | FLT1 | 2321 | http://portal.genego.com/cgi/regulation/link_info.cgi?id=-1957944205 |
| sunitinib | FLT3 | 2322 | http://portal.genego.com/cgi/regulation/link_info.cgi?id=-2071477016 |
| sunitinib | FLT4 | 2324 | http://portal.genego.com/cgi/regulation/link_info.cgi?id=-1205683485 |
| sunitinib | KDR | 3791 | http://portal.genego.com/cgi/regulation/link_info.cgi?id=-1355930151 |
| sunitinib | KIT | 3815 | http://portal.genego.com/cgi/regulation/link_info.cgi?id=-1437234975 |
| sunitinib | PDGFRA | 5156 | http://portal.genego.com/cgi/regulation/link_info.cgi?id=-1011845016 |
| sunitinib | PDGFRB | 5159 | http://portal.genego.com/cgi/regulation/link_info.cgi?id=-772113041 |
| sunitinib | RET | 5979 | http://portal.genego.com/cgi/regulation/link_info.cgi?id=-1284271834 |
| tacrine | ACHE | 43 | http://portal.genego.com/cgi/regulation/link_info.cgi?id=37647 |
| tacrolimus | PPP3CA | 5530 | http://portal.genego.com/cgi/regulation/link_info.cgi?id=-2129743934 |
| tacrolimus | PPP3CB | 5532 | http://portal.genego.com/cgi/regulation/link_info.cgi?id=-674393731 |
| tacrolimus | PPP3CC | 5533 | http://portal.genego.com/cgi/regulation/link_info.cgi?id=3344 |
| tacrolimus | PPP3R1 | 5534 | http://portal.genego.com/cgi/regulation/link_info.cgi?id=-1047849091 |
| tacrolimus | PPP3R2 | 5535 | http://portal.genego.com/cgi/regulation/link_info.cgi?id=-1821128966 |
| tadalafil | PDE5A | 8654 | http://portal.genego.com/cgi/regulation/link_info.cgi?id=27313 |
| tamoxifen | ESR1 | 2099 | http://portal.genego.com/cgi/regulation/link_info.cgi?id=-1709208646 |
| tamoxifen | ESR2 | 2100 | http://portal.genego.com/cgi/regulation/link_info.cgi?id=-1886021604 |
| tamoxifen | ABCB1 | 5243 | http://portal.genego.com/cgi/regulation/link_info.cgi?id=-564908830 |
| temsirolimus | MTOR | 2475 | http://portal.genego.com/cgi/regulation/link_info.cgi?id=-253478530 |
| teniposide | TOP2A | 7153 | http://portal.genego.com/cgi/regulation/link_info.cgi?id=-1678280008 |
| teniposide | TOP2B | 7155 | http://portal.genego.com/cgi/regulation/link_info.cgi?id=-1710051691 |
| thalidomide | FGF2 | 2247 | http://portal.genego.com/cgi/regulation/link_info.cgi?id=-950988940 |
| thalidomide | MMP2 | 4313 | http://portal.genego.com/cgi/regulation/link_info.cgi?id=-13334863 |
| thalidomide | NFKB1 | 4790 | http://portal.genego.com/cgi/regulation/link_info.cgi?id=-2010954962 |
| thalidomide | TNF | 7124 | http://portal.genego.com/cgi/regulation/link_info.cgi?id=51158 |
| thalidomide | VEGFA | 7422 | http://portal.genego.com/cgi/regulation/link_info.cgi?id=-409986014 |
| theophylline | PDE3A | 5139 | http://portal.genego.com/cgi/regulation/link_info.cgi?id=-310387782 |
| theophylline | PDE4B | 5142 | http://portal.genego.com/cgi/regulation/link_info.cgi?id=-471033730 |
| thioguanine | HPRT1 | 3251 | http://portal.genego.com/cgi/regulation/link_info.cgi?id=-95545594 |
| thioguanine | IMPDH1 | 3614 | http://portal.genego.com/cgi/regulation/link_info.cgi?id=-420502220 |
| thioguanine | PPAT | 5471 | http://portal.genego.com/cgi/regulation/link_info.cgi?id=-1202194673 |
| thioridazine | ADRA1A | 148 | http://portal.genego.com/cgi/regulation/link_info.cgi?id=-1016495387 |
| thioridazine | DRD1 | 1812 | http://portal.genego.com/cgi/regulation/link_info.cgi?id=-1011960286 |
| thioridazine | DRD2 | 1813 | http://portal.genego.com/cgi/regulation/link_info.cgi?id=-1212238106 |
| thioridazine | HTR2A | 3356 | http://portal.genego.com/cgi/regulation/link_info.cgi?id=-812220877 |
| tolcapone | COMT | 1312 | http://portal.genego.com/cgi/regulation/link_info.cgi?id=34760 |
| tolmetin | PTGS1 | 5742 | http://portal.genego.com/cgi/regulation/link_info.cgi?id=-1241986846 |
| tolmetin | PTGS2 | 5743 | http://portal.genego.com/cgi/regulation/link_info.cgi?id=-1695694131 |
| topotecan | TOP1 | 7150 | http://portal.genego.com/cgi/regulation/link_info.cgi?id=-325352321 |
| toremifene | ESR1 | 2099 | http://portal.genego.com/cgi/regulation/link_info.cgi?id=-847974905 |
| tranylcypromine | MAOA | 4128 | http://portal.genego.com/cgi/regulation/link_info.cgi?id=35802 |
| trastuzumab | ERBB2 | 2064 | http://portal.genego.com/cgi/regulation/link_info.cgi?id=-802327007 |
| trazodone | ADRA1A | 148 | http://portal.genego.com/cgi/regulation/link_info.cgi?id=-1520721239 |
| trazodone | ADRA2A | 150 | http://portal.genego.com/cgi/regulation/link_info.cgi?id=-1324942922 |
| trazodone | HRH1 | 3269 | http://portal.genego.com/cgi/regulation/link_info.cgi?id=-1735015732 |
| trazodone | HTR1A | 3350 | http://portal.genego.com/cgi/regulation/link_info.cgi?id=-405059117 |
| trazodone | HTR2A | 3356 | http://portal.genego.com/cgi/regulation/link_info.cgi?id=-1406632658 |
| trazodone | SLC6A4 | 6532 | http://portal.genego.com/cgi/regulation/link_info.cgi?id=-1196933770 |
| valproic acid | HDAC6 | 10013 | http://portal.genego.com/cgi/regulation/link_info.cgi?id=-399679227 |
| vardenafil | PDE5A | 8654 | http://portal.genego.com/cgi/regulation/link_info.cgi?id=27319 |
| vinblastine | TUBB2A | 7280 | http://portal.genego.com/cgi/regulation/link_info.cgi?id=-1467613493 |
| vinblastine | TUBB | 203068 | http://portal.genego.com/cgi/regulation/link_info.cgi?id=-1467613493 |
| vinblastine | TUBB2B | 347733 | http://portal.genego.com/cgi/regulation/link_info.cgi?id=-1467613493 |
| vorinostat | HDAC1 | 3065 | http://portal.genego.com/cgi/regulation/link_info.cgi?id=-487934547 |
| vorinostat | HDAC2 | 3066 | http://portal.genego.com/cgi/regulation/link_info.cgi?id=-2049266121 |
| vorinostat | HDAC3 | 8841 | http://portal.genego.com/cgi/regulation/link_info.cgi?id=-1332107678 |
| vorinostat | HDAC4 | 9759 | http://portal.genego.com/cgi/regulation/link_info.cgi?id=-554709601 |
| vorinostat | HDAC6 | 10013 | http://portal.genego.com/cgi/regulation/link_info.cgi?id=-1764042978 |
| vorinostat | HDAC7 | 51564 | http://portal.genego.com/cgi/regulation/link_info.cgi?id=-1959619249 |
| vorinostat | HDAC8 | 55869 | http://portal.genego.com/cgi/regulation/link_info.cgi?id=-644654487 |
| yohimbine | ADRA2A | 150 | http://portal.genego.com/cgi/regulation/link_info.cgi?id=36810 |
| yohimbine | ADRA2B | 151 | http://portal.genego.com/cgi/regulation/link_info.cgi?id=-537881176 |
| yohimbine | ADRA2C | 152 | http://portal.genego.com/cgi/regulation/link_info.cgi?id=36809 |
