# Supplementary material for: Prospective Molecular Profiling of Canine Cancers Provides a Clinically Relevant Comparative Model for Evaluating Personalized Medicine (PMed) Trials
Source: PLoS One. 2014 Mar 17;9(3):e90028. doi: 10.1371/journal.pone.0090028 (PMC3956546; doi:10.1371/journal.pone.0090028)
Supplement: Table S3 — Biomarker rules. Thirty-four biomarker rules are shown that match 20 FDA approved drugs to 22 targets according to sensitivity or resistance alongside supporting evidence for these interactions. (DOCX) [file pone.0090028.s004.docx]

| **Entrez ID** | **Entrez Symbol** | **Range Min** | **Range Max** | **Resistance** | **DRUG** | **Evidence** |
| --- | --- | --- | --- | --- | --- | --- |
| 100 | ADA |  | -3 | 0 | cytarabine | <http://www.ncbi.nlm.nih.gov/pubmed/15632314> |
| 440 | ASNS |  | -3 | 0 | pegaspargase | <http://www.ncbi.nlm.nih.gov/pubmed/18852115> |
| 440 | ASNS |  | -3 | 0 | asparaginase | <http://www.ncbi.nlm.nih.gov/pubmed/18852115> |
| 1633 | DCK | 3 |  | 0 | gemcitabine | <http://www.ncbi.nlm.nih.gov/pubmed/20043109> |
| 1890 | TYMP | 3 |  | 0 | capecitabine | <http://www.ncbi.nlm.nih.gov/pubmed/18765464> |
| 2067 | ERCC1 | 3 |  | 1 | cisplatin | <http://www.ncbi.nlm.nih.gov/pubmed/19574766> |
| 2067 | ERCC1 | 3 |  | 1 | carboplatin | <http://www.ncbi.nlm.nih.gov/pubmed/19574766> |
| 2067 | ERCC1 | 3 |  | 1 | oxaliplatin | <http://www.ncbi.nlm.nih.gov/pubmed/19574766> |
| 2099 | ESR1 | 3 |  | 0 | medroxyprogesterone | <http://www.drugbank.ca/drugs/DB00603> |
| 3559 | IL2RA | 3 |  | 0 | aldesleukin | [http://www.drugbank.ca/drugs/DB00041 ; http://www.ncbi.nlm.nih.gov/pubmed/19692326](http://www.drugbank.ca/drugs/DB00041%20;%20http://www.ncbi.nlm.nih.gov/pubmed/19692326) |
| 4255 | MGMT |  | -3 | 0 | temozolomide | <http://www.ncbi.nlm.nih.gov/pubmed/16541434> |
| 4255 | MGMT | 3 |  | 1 | temozolomide | <http://www.ncbi.nlm.nih.gov/pubmed/20428759> |
| 4363 | ABCC1 | 3 |  | 1 | doxorubicin | <http://www.ncbi.nlm.nih.gov/pubmed/11172691> |
| 5241 | PGR | 3 |  | 0 | medroxyprogesterone | <http://www.drugbank.ca/drugs/DB00603> |
| 6240 | RRM1 |  | -3 | 0 | gemcitabine | <http://www.ncbi.nlm.nih.gov/pubmed/20443070> |
| 6241 | RRM2 |  | -3 | 0 | gemcitabine | <http://www.ncbi.nlm.nih.gov/pubmed/19002265> |
| 6241 | RRM2 |  | -3 | 0 | hydroxyurea | <http://www.ncbi.nlm.nih.gov/pubmed/7882331> |
| 6257 | RXRB | 3 |  | 0 | bexarotene | <http://www.drugbank.ca/drugs/DB00307> |
| 6678 | SPARC | 3 |  | 0 | paclitaxel albumin-bound | <http://www.ncbi.nlm.nih.gov/pubmed/18766004> |
| 7298 | TYMS | 3 |  | 1 | fluorouracil | <http://www.ncbi.nlm.nih.gov/pubmed/17425594> |
| 7298 | TYMS | 3 |  | 1 | capecitabine | <http://www.ncbi.nlm.nih.gov/pubmed/17425594> |
| 7421 | VDR | 3 |  | 0 | calcitriol | <http://www.drugbank.ca/drugs/DB00136> |
| 9429 | ABCG2 | 3 |  | 1 | topotecan | <http://www.ncbi.nlm.nih.gov/pubmed/20019844> |
| 332 | BIRC5 |  | -3 | 0 | bortezomib | <http://www.ncbi.nlm.nih.gov/pubmed/20096120> |
| 2067 | ERCC1 |  | -3 | 0 | carboplatin | <http://www.ncbi.nlm.nih.gov/pubmed/18977553> |
| 2067 | ERCC1 |  | -3 | 0 | cisplatin | <http://www.ncbi.nlm.nih.gov/pubmed/18977553> |
| 100 | ADA | 3 |  | 1 | cytarabine | <http://www.ncbi.nlm.nih.gov/pubmed/15632314> |
| 2030 | SLC29A1 |  | -3 | 1 | cytarabine (Resistance) | <http://www.ncbi.nlm.nih.gov/pubmed/15632314> |
| 2030 | SLC29A1 | 3 |  | 0 | cytarabine | <http://www.ncbi.nlm.nih.gov/pubmed/15632314> |
| 1646 | AKR1C2 | 3 |  | 1 | doxorubicin | <http://www.ncbi.nlm.nih.gov/pubmed/19440163> |
| 8644 | AKR1C3 | 3 |  | 1 | doxorubicin | <http://www.ncbi.nlm.nih.gov/pubmed/19440163> |
| 2067 | ERCC1 |  | -3 | 0 | oxaliplatin | <http://www.ncbi.nlm.nih.gov/pubmed/18977553> |
| 5887 | RAD23B | 3 |  | 0 | bortezomib | <http://www.ncbi.nlm.nih.gov/pubmed/20308564> |
| 5887 | RAD23B | 3 |  | 0 | vorinostat | <http://www.ncbi.nlm.nih.gov/pubmed/20308564> |

**Supplementary Table 3: Biomarker Rules**
