# Supplementary material for: Prospective Molecular Profiling of Canine Cancers Provides a Clinically Relevant Comparative Model for Evaluating Personalized Medicine (PMed) Trials
Source: PLoS One. 2014 Mar 17;9(3):e90028. doi: 10.1371/journal.pone.0090028 (PMC3956546; doi:10.1371/journal.pone.0090028)
Supplement: Table S4 — Response signature drugs. The subset of 107 CMAP drugs refined based on literature support and used to match drugs based on disease genotypes. (DOCX) [file pone.0090028.s005.docx]

**Supplementary Table 4: Response Signature Drugs**

| **Generic Drug Name** |
| --- |
| acetylsalicylic acid |
| amiloride |
| aminoglutethimide |
| aminophylline |
| amitriptyline |
| azacitidine |
| azathioprine |
| bendroflumethiazide |
| brinzolamide |
| bumetanide |
| carbamazepine |
| celecoxib |
| chlorpromazine |
| chlorpropamide |
| clotrimazole |
| clozapine |
| colchicine |
| cyclosporin |
| dantrolene |
| daunorubicin |
| decitabine |
| deferoxamine |
| dexamethasone |
| diclofenac |
| diflunisal |
| digoxin |
| disulfiram |
| dopamine |
| doxorubicin |
| doxycycline |
| estradiol |
| etodolac |
| etoposide |
| exemestane |
| felodipine |
| fenoprofen |
| finasteride |
| fludrocortisone |
| fluphenazine |
| flurbiprofen |
| flutamide |
| fluvastatin |
| fulvestrant |
| furosemide |
| gefitinib |
| haloperidol |
| hydrochlorothiazide |
| iloprost |
| imatinib |
| indomethacin |
| irinotecan |
| isocarboxazid |
| ketoprofen |
| ketorolac |
| lansoprazole |
| leflunomide |
| letrozole |
| lomustine |
| lovastatin |
| mefenamic acid |
| megestrol |
| mercaptopurine |
| mesalamine |
| metformin |
| methotrexate |
| milrinone |
| minocycline |
| mitoxantrone |
| nabumetone |
| naproxen |
| nifedipine |
| omeprazole |
| orlistat |
| oxaprozin |
| paclitaxel |
| pentamidine |
| phenelzine |
| phentolamine |
| piroxicam |
| prazosin |
| prednisolone |
| prochlorperazine |
| propylthiouracil |
| raloxifene |
| ramipril |
| reserpine |
| simvastatin |
| sirolimus |
| sodium phenylbutyrate |
| sulfasalazine |
| tacrine |
| tacrolimus |
| thalidomide |
| thioguanine |
| thioridazine |
| tolbutamide |
| tolmetin |
| topiramate |
| tranylcypromine |
| trazodone |
| trifluoperazine |
| valproic acid |
| vardenafil |
| verapamil |
| vinblastine |
| vorinostat |
| yohimbine |
