# Supplementary material for: Prospective Molecular Profiling of Canine Cancers Provides a Clinically Relevant Comparative Model for Evaluating Personalized Medicine (PMed) Trials
Source: PLoS One. 2014 Mar 17;9(3):e90028. doi: 10.1371/journal.pone.0090028 (PMC3956546; doi:10.1371/journal.pone.0090028)
Supplement: Table S5 — Sensitivity profile drugs. The subset of 11 drugs selected from the NCI-60 list COMPARE database and matched to gene expression signatures. (DOCX) [file pone.0090028.s006.docx]

**Supplementary Table 5: Sensitivity Profile Drugs**

| **Generic Drug Name** |
| --- |
| carmustine |
| cisplatin |
| daunorubicin |
| doxorubicin |
| etoposide |
| lomustine |
| paclitaxel |
| piperazine |
| thioguanine |
| thiotepa |
| vinblastine |
| carmustine |
| cisplatin |
| daunorubicin |
| doxorubicin |
| etoposide |
| lomustine |
| paclitaxel |
| piperazine |
| thioguanine |
